# Novel therapeutic strategies for ccRCC through integration of systemic drug combinations and oncolytic adenovirus immunotherapy

**DOI:** 10.3389/fimmu.2026.1760889

**Published:** 2026-08-14

**Authors:** Michaela Feodoroff, Isabel Mogollon, Firas Hamdan, Tamara J. Luck, Romika Kumari, Paolo Bottega, Janita Sandberg, Erika Romppanen, Lassi Paavolainen, Minna Malmstedt, Patrick Penttilä, Olli Kallioniemi, Tuomas Mirtti, Antti Rannikko, Mikaela Grönholm, Vincenzo Cerullo, Vilja Pietiäinen

**Affiliations:** 1 Laboratory of Immunovirotherapy, Drug Research Program, University of Helsinki Faculty of Pharmacy, Helsinki, Finland; 2 Institute for Molecular Medicine Finland (FIMM), Helsinki Institute for Life Sciences (HiLIFE), University of Helsinki, Helsinki, Finland; 3 TRIMM, Translational Immunology Research Program, University of Helsinki, Helsinki, Finland; 4 Drug Delivery, Drug Research Program, Division of Pharmaceutical Biosciences, Faculty of Pharmacy, University of Helsinki, Helsinki, Finland; 5 iCAN Digital Precision Cancer Medicine Flagship, University of Helsinki, Helsinki, Finland; 6 Department of Urology, Helsinki University Hospital (HUS), Helsinki, Finland; 7 Research Program in Systems Oncology, Faculty of Medicine, University of Helsinki, Helsinki, Finland; 8 Finnish Cancer Institute (FCI), Helsinki, Finland; 9 Department of Pathology, Helsinki University Hospital (HUS), Helsinki, Finland; 10 Department of Molecular Medicine and Medical Biotechnology and CEINGE, Naples University 24 Federico II, Naples, Italy

**Keywords:** ccRCC, drug screening, oncolytic adenovirus therapy, PDCs, targeted therapy

## Abstract

**Introduction:**

Clear cell renal cell carcinoma (ccRCC) remains a significant therapeutic challenge due to its biological heterogeneity, variable clinical behavior, and limited efficacy of single-agent systemic therapies. Combination strategies targeting multiple tumor-promoting pathways may improve therapeutic outcomes.

**Methods:**

We evaluated combination treatment strategies integrating targeted systemic drugs with oncolytic adenovirus therapy using representative patient-derived cancer cell (PDC) models. Anti-tumor efficacy and immune-mediated cytotoxicity were assessed to investigate treatment responses across different patient-derived models.

**Results:**

Combination treatment enhanced anti-tumor activity compared with either monotherapy alone and promoted immune-mediated cytotoxicity involving CD8+ T cells and natural killer (NK) cells. Treatment responses varied across the three PDC models, reflecting interpatient heterogeneity and suggesting potential value for biomarker-guided patient stratification.

**Discussion:**

These findings demonstrate the potential of combining targeted therapy with oncolytic virotherapy to overcome current therapeutic limitations in ccRCC. Moreover, the use of advanced three-dimensional human-relevant PDC models provides a scalable platform for evaluating combination therapies, supporting personalized treatment strategies, and facilitating the development of therapeutic approaches applicable to ccRCC and other cancer types.

## Introduction

Kidney cancer remains among the ten most frequently diagnosed cancers worldwide, with ccRCC accounting for about 80% of cases. Most tumors are discovered incidentally, and patients often exhibit minimal or no symptoms at the time of diagnosis. However, approximately 20% of patients have already developed metastases at the time of diagnosis, which substantially worsens both prognosis and treatment response.

ccRCC is characterized by profound metabolic reprogramming, largely driven by oncogenic alterations such as loss of the VHL gene (1, 2). This loss activates hypoxia-inducible factors (HIFs), which in turn stimulate transcription of vascular endothelial growth factor (VEGF) genes (3). The resulting cascade promotes vascular permeability, tumor cell migration, and ultimately cancer progression. These molecular events are accompanied by extensive metabolic alterations, including enhanced glycolysis, dysregulated lipid metabolism, and altered glutamine utilization. Such changes not only drive tumor growth but also present potential vulnerabilities for targeted therapeutic intervention. Although targeted therapies such as cabozantinib, used alone or in combination with nivolumab, pazopanib and sunitinib, have improved clinical outcomes, their effectiveness is often limited by drug resistance and the rarity of complete remissions. This underscores the need for innovative strategies, such as immuno-oncology approaches, to complement current treatments.

In this context, immune checkpoint inhibitor (ICI)-based therapies have emerged as a cornerstone of systemic treatment in clear cell renal cell carcinoma, either as monotherapy or in combination with VEGF-targeted agents (4, 5). These strategies are supported by the interplay between angiogenesis and immune suppression within the tumor microenvironment (TME), where VEGF signaling contributes not only to vascular remodeling but also to immune evasion, thereby reinforcing the rationale for combination approaches aimed at achieving more durable and potentially disease-controlling responses (5). Despite these advances, clinical benefit remains heterogeneous, with a substantial proportion of patients failing to achieve durable responses. This variability is partly attributed to primary and adaptive resistance mechanisms, as well as the lack of robust predictive biomarkers for treatment stratification. Although several candidate biomarkers have been proposed to predict treatment response in renal cell carcinoma, spanning molecular (e.g., kidney injury molecule-1 (KIM-1), PD-L1 expression, tumor mutational burden (TMB)), immune-related (e.g., patterns of immune cell infiltration), and histopathological features, their predictive performance remains limited (6, 7). Consequently, there is growing interest in integrated approaches that combine TME characteristics, genomic alterations, and immune-related gene expression signatures to improve patient selection and optimize therapeutic outcomes.

Oncolytic adenoviruses are genetically engineered viruses that selectively replicate and destroy cancer cells while sparing healthy tissue, taking advantage of the impaired antiviral defenses characteristic of many tumor cells (8). Beyond their intrinsic oncolytic activity, modern generations of these viruses are increasingly developed as therapeutic platforms capable of delivering immunomodulatory payloads directly into the TME, thereby promoting immunogenic cell death and overcoming resistance mechanisms that otherwise suppress standard monotherapy efficacy and antitumor immune responses (8–10). As infected tumor cells are destroyed, they release both viral and tumor antigens, along with the therapeutic transgene products expressed by the virus, which amplify immune responses against malignant cells (11, 12). This strategy distinguishes them from conventional, purely oncolytic viruses by coupling direct tumor destruction with targeted immune stimulation, representing a major advancement that provides therapeutic versatility, tumor selectivity, and improved safety. The clinical improvement of armed oncolytic adenoviruses is exemplified by TILT-123 (13), ONCOS-102 (14), and LOAd703 (15), which are evaluating payload-expressing adenoviruses across a broad spectrum of solid tumors. The progress of these trials underscores a rapidly expanding clinical and translational interest in virus-mediated immune modulation as a novel therapeutic strategy in oncology. In the context of ccRCC, oncolytic adenoviruses are particularly promising due to their potential synergy with various therapeutic agents and their ability to target resistant cancer cell populations that frequently persist after standard monotherapy. In this study, we employed the oncolytic adenovirus AdCab as a delivery vehicle for an Fc-fusion peptide engineered to activate immune effector cells within the TME, thereby enhancing antitumor immunity in ccRCC.

We hypothesized that combining oncolytic adenoviruses with metabolic drugs that disrupt tumor energetics could enhance viral replication, immune cell infiltration, and tumor cell sensitivity to the drugs, thereby promoting additive/synergistic cancer cell destruction (16, 17). This study investigates the combined use of systemic drug therapy and virus-mediated immunotherapy, with both approved and investigational agents, to improve therapeutic options for ccRCC. To bridge preclinical findings with clinical applications, this approach is tested in representative three-dimensional (3D) ccRCC patient-derived tumor models, providing a robust preclinical framework for future translational development.

Our results reveal that the metabolic stress induced by inhibitors of key enzymes, such as NAMPT inhibitor daporinad, which depletes NAD+ pools and impairs cancer cell survival, can amplify the cytotoxic effects of viral oncolysis. Metabolic modulation can beneficially remodel the TME, enhancing the activation and recruitment of immune cells, such as CD8^+^ T cells and NK cells, thereby strengthening the overall anti-tumor activity. Collectively, targeting the metabolic vulnerabilities of ccRCC in conjunction with oncolytic virotherapy offers a rational, multifaceted strategy to overcome therapeutic resistance and improve clinical outcomes.

## Materials and methods

### Cell lines

786-O human clear cell renal adenocarcinoma cell-line (American Type Culture Collection (ATCC)) was maintained in RPMI (Roswell Park Memorial Institute, Gibco, Cat#21875034) medium supplemented with 10% fetal bovine serum (FBS) (Thermo Fisher Scientific, Cat#10500064), 1% GlutaMAX (Thermo Fisher Scientific, Cat#35050038), and 1% penicillin–streptomycin (10,000 U/mL) (Gibco, Cat#15140122) at 37 °C in an atmosphere of 5% CO_2_.

### Patient-derived cell cultures

PDCs from three clinically diagnosed ccRCC patients (Patient 1, Patient 2 and Patient 3) (**Supplementary Table 1**) were isolated as previously described (18–20) and cultured in a 3:1 (v/v) mixture of Ham´s F-12 Nutrient mix (Gibco, Cat#11765054) and DMEM (Gibco, Cat#11965092). The medium was supplemented with 5% FBS, 8,4 ng/mL Choleratoxin (Sigma, Cat#C8052), 0.4 µg/mL hydrocortisone (Sigma, Cat#H4001), 10 ng/mL epidermal growth factor (Corning, Cat#354052), 24 µg/mL Adenine (Sigma, Cat#A8626), 5 µg/mL insulin (Sigma, 91077C) and 10 µM of Y-27632 RHO kinase inhibitor (Sigma, Cat#SCM075). Culture surfaces were coated with 2% Matrigel prior to cell seeding.

### Whole exome sequencing

Whole exome sequencing (WES) of the tumor tissue and corresponding PDC cultures were performed as follows: 50 ng of gDNA was processed according to Twist Library Preparation EF 2.0 with Enzymatic Fragmentation DOC-001239 REV 1.0 and Twist Target Enrichment Protocol DOC-001273 REV 3.0 manual (Twist Bioscience, San Francisco, CA, USA). The following adapters were used for ligation: PerkinElmer NEXTFLEX unique dual index (UDI), length 10 bp, 5 µl of 10 µM (PerkinElmer, Waltham, MA, USA). Library quantification and quality check were performed using LabChip GX Touch HT High Sensitivity assay (PerkinElmer, Waltham, MA, USA) and Qubit Broad Range DNA Assay (Thermo Fisher Scientific, Waltham, MA, USA). Libraries were pooled to 8-plex reactions according to concentration (Qubit BR assay). The exome enrichment was performed using either Twist Core or MedExome. The captured library pools were quantified for sequencing using QuantStudio 5 Colibri Library Quantification kit (Thermo Fisher Scientific, Waltham, MA, USA) and LabChip GX Touch HT High Sensitivity assay (PerkinElmer, Waltham, MA, USA). Sequencing was performed via the Illumina NovaSeq 6000 system using S4 flow cell (Illumina, San Diego, CA, USA) and v1.5 chemistry. Read length for the paired-end run was 2x 99 bp or 2x 101 bp. Illumina Dragen analysis pipeline v3.9 or 4.0 was used for the analysis.

DRAGEN somatic mode VCFs were annotated with ANNOVAR (21) and filtered to retain only non-synonymous exonic and splice-site variants passing the default DRAGEN filters (PASS in the VCF) and with a VAF >= 5%. From the filtered somatic mutations “functionally relevant” mutations were identified by the following criteria, i) Pathogenicity (predicted to be “Pathogenic” or “Likely pathogenic”) based on the ACMG/AMP 2015 guidelines for clinical interpretation of genetic variants by InterVar (22), and/or ii) AMP/ASCO/CAP guidelines classification of a Tier ≤ 3 mutation by the Personal Cancer Genome Reporter (PCGR) (version 2.2.5 tumor primary site “kidney”) (23). Functionally relevant mutations were further considered “clinically relevant” when associated with disease diagnosis, predisposition, prognosis, and/or drug response by the PCGR. The heatmap shows the TMB per megabase based on all coding non-silent somatic PASS mutations and further visualizes the subset of mutations which are i) affecting genes recurrently mutated in 2 or more patients, and/or ii) determined to be functionally and/or clinically relevant by InterVar and/or PCGR. Note that the first criterion i) only considers non-hypermutated samples with less than 1000 filtered somatic mutations to keep the heatmap legible.

### Oncolytic adenovirus

The human oncolytic 5/3 serotype adenovirus used in this study has a 24-bp deletion in the E1A region, generated as previously described (24). AdCab also carries a replacement of the gp19K+7.1K region in the E3 gene with a transgene encoding for a cross-hybrid IgGA Fc-fusion peptide as reported (25).

### Drug sensitivity and resistance testing

Prior to 3D drug sensitivity and resistance testing (DSRT), the cells stored in liquid nitrogen were thawed and cultured for a maximum of 1–3 passages to ensure they were in an optimal growth state. A fresh cell suspension was prepared and dispensed at a density of 1000 cells per well into pre-drugged u-bottom 384-well plates (Corning, #3830) or on viability plates with no drugs. All drugs (**Supplementary Table 2**) were annotated for oncology status by querying openFDA (FDA drug-label and Drugs@FDA data), the European Medicines Agency (EMA) medicines report, ClinicalTrials.gov (v2 API, for highest clinical phase), and the Open Targets Platform (maximum clinical phase/approval flag), and were subsequently tested in five different concentrations with a 10,000-fold concentration range in a total well volume of 25 μL, following an established protocol (18). Cells were seeded using the MultiFlo FX RAD (Random Access Dispensing) system (BioTek), equipped with a 5 μL dispensing cassette (Agilent BioTek, Cat#1260016). AdCab (infected condition) or fresh culture medium (uninfected MOCK condition) was dispensed into designated wells using MANTIS Liquid Dispenser (Formulatrix), Low Volume Silicone Chips (Formulatrix) and Mantis Liquid Handler Control Software (firmware version 4.20.6) at the time of cell seeding. The plates were centrifuged at 300 g for 5 minutes and incubated at 37 °C in a humidified atmosphere with 5% CO_2_ for 72 h. At the endpoint of the experiment, cell viability was assessed using a luminescence-based metabolic assay. CellTiter-Glo^®^ 2.0 reagent (CTG, Promega, Cat#G924) was added to each well (15 μL per well) using the MultiFlo FX RAD dispenser. The plates were shaken for 5 minutes, centrifuged at 300 g for 3 minutes, and incubated at room temperature for 30 minutes to allow the luminescent signal to stabilize. Luminescence was then recorded using the PHERAstar FS microplate reader (BMG Labtech). Data was analyzed using Breeze software if not otherwise mentioned (26).

### Drug response evaluation

Statistical analysis of patient-specific drug responses was performed as follows:

Out of the 26 drugs tested, we identified 16 that were effective, i.e. achieved a mean DSS of at least 5, in at least one patient (1/2/3) and condition (MOCK/AdCab). The Kruskal-Wallis test was used to identify 11/16 drugs with significant patient-specific responses (p ≤ 0.1 in MOCK and/or AdCab). In addition, we quantified the signal-to-noise ratio of these 26 drugs and found 10/26 with a standard deviation of the mean DSS across patients (biological replicates) at least 1.5 times higher than the standard deviation of the DSS across technical replicates. All 10 of these had also returned significant p values (p ≤ 0.1) in the Kruskal-Wallis test and were therefore deemed ‘significantly heterogeneous’ across patients in at least one (MOCK/AdCab) condition.

Drug targets were identified using the Therapeutic Target Database (TTD) (27) and mapped to immune-related genesets using HALLMARKS (28) and REACTOME (29) gene set collections from MsigDB (30) (msigdb, hallmarks, reactome), resulting in six of the 10 drugs being successfully mapped to eight different immune-related gene sets.

### Synergy analysis

The SynergyFinder R package (v3.20.0) (31) was used to perform synergy analysis of 26 DSRT compounds +/- AdCab for the 786-O cell line (two replicates) and three PDC models (Patient 1/2/3). First, percent viability was calculated from raw CTG values relative to plate-specific positive and negative controls. For each uninfected Mock plate, using same plate average BzCl signal for background subtraction and same plate average DMSO signal to scale to 100% viability, percent viability is calculated as:


$$Viability=\frac{\left(CTG_{sample}-{\bar{CTG}}_{BzCl.mock}\right)}{\left({\bar{CTG}}_{DMSO.mock}-{\bar{CTG}}_{BzCl.mock}\right)}\cdot 100$$

For infected AdCab plates, same plate BzCl signal was similarly used for background subtraction and average Mock DMSO signal to scale to 100% viability:


$$Viability=\frac{\left(CTG_{sample}-{\bar{CTG}}_{BzCl.adcab}\right)}{\left({\bar{CTG}}_{DMSO.mock}-{\bar{CTG}}_{BzCl.adcab}\right)}\cdot 100$$

These viability measures were fed into SynergyFinder’s ReshapeData(), CalculateSynergy() and CalculateSensitivity() functions with default parameters to obtain Highest Single Agent (HSA) synergy scores, individual drug and virus Relative Inhibition (RI) and Combination Sensitivity Score (CSS) values. The results were visualized using ggplot2.

### PBMC purification

Peripheral blood mononuclear cells (PBMCs) were obtained from buffy coats of healthy donors supplied by the Finnish Red Cross through a two-step density gradient separation using Ficoll (Sigma, Cat#GE17-1440-02). The cells were isolated by centrifuging twice, initially at 400 g for 30 minutes, followed by a second centrifugation at 400 g for 10 minutes, both at room temperature. To eliminate residual red blood cells, samples were treated with ACK lysis buffer (Gibco, Cat#11509876) and centrifuged. The resulting PBMCs were then suspended in supplemented RPMI medium.

### NK cell isolation

Human NK cells were isolated from purified PBMCs using MojoSort™ Human NK Cell Isolation Kit (BioLegend Cat#480053) according to the manufacturer’s protocol. Diluted MojoSort Buffer (5X) (Cat#480017) was used for all washing steps as indicated in the protocol and MojoSort Magnet 5mL (Cat#480019) was used to separate magnetically labeled cells. The magnetic separation was repeated for a total of two separations to increase the final yield.

### Flow cytometry

The following pre-labeled antibodies were used for sample stainings:

APC anti-human CD274 (B7-H1, PD-L1, Clone 29E.2A3, BioLegend Cat#329708), PE/Cyanine7 anti-human CD3 (Clone HIT3a, BioLegend Cat#300316), Alexa Fluor 700 CD3 (Clone UCHT1, eBioscience Cat#56-0038-82), eFluor 506 CD4 (Clone RPA-T4, eBioscience Cat#69-0049-41), PE/Cyanine5 anti-human CD8 (Clone SK1, BioLegend Cat#344770), Pacific Blue anti-human CD279 (Clone EH12.2H7, BioLegend Cat#329916), FITC anti-human CD56 (Clone MEM-188, BioLegend, CAT#304604), PerCP anti-human CD107a (Clone H4A3, BioLegend, CAT# 328641), Human TruStain FcX (BioLegend Cat#422302).

PDCs were plated at 1 × 10^4^ cells/well on 96-well plates and exposed to AdCab virus at a multiplicity of infection (MOI) 10 and/or drug compounds at selected concentrations. Drugs were manually added to each well from compound aliquots obtained from the FIMM High Throughput Biomedicine Unit (HiLIFE, University of Helsinki). Effector cells were plated 48 h after PDC target cell seeding and incubated for 6 h prior to staining and data acquisition using BD LSRFortessa (BD Bioscience). All data was analyzed using FlowJo software version 9.

### Immunofluorescence staining

For immunofluorescence staining of 786-O cancer cells in a monolayer, cells were cultured on 8-well Chamber Slides (Nunc; Lab-Tek) with removable wells (Thermo Fisher Scientific, CAT#154534) and infected with AdCab at an MOI of 10 prior to staining. Live-cell dyes including Hoechst 33342 nucleic acid stain (Thermo Scientific, CAT#H3570) and Calcein AM Green (Thermo Fisher Scientific, CAT#C34852), were applied for 30 minutes at 37 °C in 5% CO_2_ prior to fixation using 4% paraformaldehyde and permeabilization with 0.1% Triton X-100. Cells were then stained with FITC Adenovirus Hexon Monoclonal Antibody (Thermo Fisher Scientific, CAT#MA17329) at a 1:100 dilution for 60 minutes on a plate rocker at room temperature. After final washing steps, images were acquired using the Invitrogen EVOS M7000 Imaging System (Thermo Fisher Scientific).

Before high-content 3D spheroid imaging, 786-O cells were labeled with CellTracker™ Orange CMRA (Thermo Fisher Scientific, CAT#C34551) and incubated for 30 minutes at 37 °C in 5% CO_2_ prior to seeding on 384-well plates. After 48 hours of spheroid formation and drug treatment, PBMCs were labeled with Calcein AM Green (Thermo Fisher Scientific, CAT#C34852) for 30 minutes at 37 °C in 5% CO_2_ and then added to the spheroid cultures. Nuclear staining was performed using SPY650-DNA dye (Spirochrome AG) before fixation with 4% paraformaldehyde. Following final PBS washes, images were acquired using the Opera Phenix™ high-content confocal microscope (Revvity).

### Confocal high-content microscopy and image analysis

All images were acquired using PerkinElmer Opera Phenix™ (Revvity) high-content confocal microscope with 20X water immersion objective, NA 1.0. Raw 3D image stacks (0.299 × 0.299 × 2.5 µm voxel size, 45 z-slices, 2160 × 2160 pixels) were quality-checked in Harmony (Revvity) and BIAS (Single Cell Technologies Ltd.) to assess signal intensity and staining penetration. Images were exported as 3D TIFFs and processed with custom Python pipelines performing channel merging, normalization, and contrast enhancement, followed by deep learning–based whole-spheroid and single-cell segmentation. Segmentation parameters were optimized via Bayesian optimization, and outputs were inspected in Napari. The workflow followed the principles of the MorphoNavigator-3D framework, developed in-house (32), which integrates 3D image preprocessing, segmentation, and high-dimensional phenotyping of tumor–immune spheroids. Sphericity measures were performed according to how close the spheroid is to a perfect sphere-like shape, where values close to 1 reflect a perfect sphere and values close to 0 indicate irregular, elongated or flattened shapes. For cell scattering (nearest-neighbor distance, NND) cancer cell centroids were extracted and converted into physical units. Mean nearest-neighbor distances (NNDs) were computed per spheroid using a k-d tree. To normalize for spheroid size, mean NNDs were divided by the equivalent sphere radius, yielding a scale-independent scattering index. To quantify the spatial organization of cancer cells and PBMCs within spheroids, we computed a panel of complementary 3D spatial metrics from segmented cell centroids (converted to micrometers using voxel dimensions 0.299 × 0.299 × 2.5 µm). Analyses were performed per spheroid field of view (Well_FOV) and aggregated by drug condition. PDC and PBMC interactions were determined according to the following criteria, where high interaction proportions reflect many cancer-PBMC cell pairs at close distances (< 12 µm) indicating strong spatial mixing, and low interaction proportions reflect cancer cells and PBMCs present in a specific region but not close enough to interact indicating segregation or sparsity. Regional comparison of the spheroid center and periphery was performed by first volume-normalizing all regions thereby generating differences that are not due to sphere size but reflect true biological/phenotypic differences in how cells localize and engage across treatment conditions.

### Statistical data analysis

Statistical analysis for the data shown in bar plots was performed using GraphPad Prism 10.6 (GraphPad Software, USA). Data were analyzed using one-way ANOVA or two-way ANOVA with n≥3. Levels of significance were set at *p<0.05, **p<0.01, ***p<0.001 and ****p<0.0001. Error bars are representative of the standard error of mean (SEM). For drug specific curves, the drug concentrations were first log-transformed and then subjected to nonlinear regressions of log(agonist) versus response-variable slope (four parameters) analysis. Raw detection values generated using luminescence counts obtained using the CellTiter-Glo assay were used as input data for DSRT analysis.

## Results

**Figure 1:**
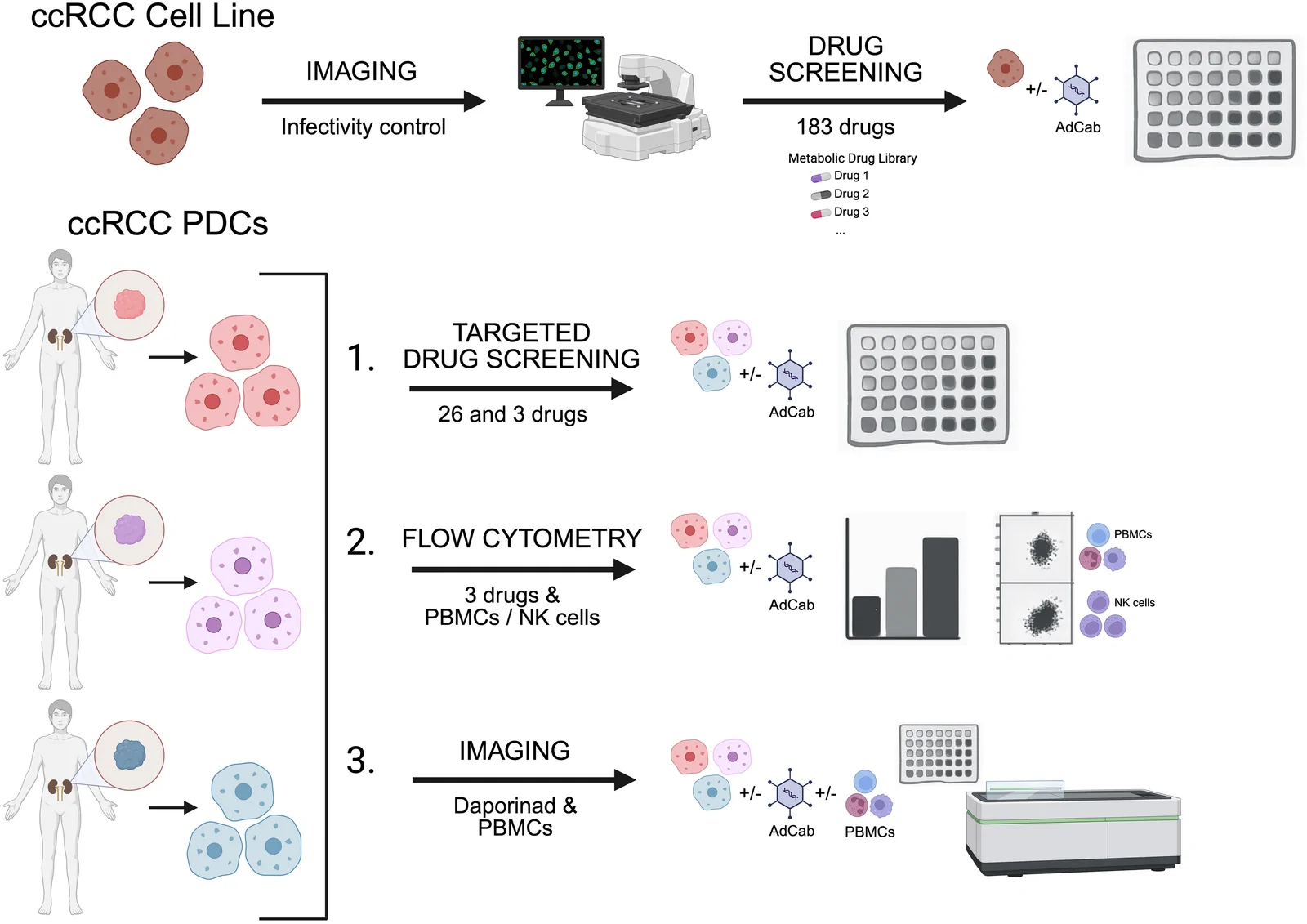
Schematic overview of the translational framework evaluating combined systemic and oncolytic immunotherapy in ccRCC. The schematic illustrates the translational workflow integrating drug screening, flow cytometry and imaging to assess combination therapeutic strategies for ccRCC using 786-O cell line and three PDC models.

In this study, we combined drug screening with oncolytic adenovirus-based immunotherapy to identify synergistic opportunities for therapeutic development. The schematic overview of the study design illustrates the workflow used to evaluate novel therapeutic strategies for ccRCC (**Figure 1**). The 786-O ccRCC cell line was initially used for high-throughput screening of a metabolic drug library comprising 182 compounds to identify drug classes with potential effects on tumor cell viability, metabolic regulation, and immune-related pathways. Subsequently, three ccRCC PDC models were used for targeted drug screening, in which candidate compounds showing enhanced cytotoxicity and immune modulation were selected for further functional validation by flow cytometry to assess immune cell activation. Finally, the same three PDC models were analyzed using imaging-based approaches in the presence of daporinad and human PBMCs to visualize cross-cell type interactions and evaluate treatment-induced alterations in tumor-immune spatial dynamics. Overall, this integrated workflow defined a translational framework that bridges drug discovery, viral immunotherapy, and immune interaction profiling, thereby supporting the rational development and evaluation of combination therapies for ccRCC.

### Oncolytic fitness of AdCab and sensitivity of 786-O cells to drug compounds targeting metabolic pathways

**Figure 2:**
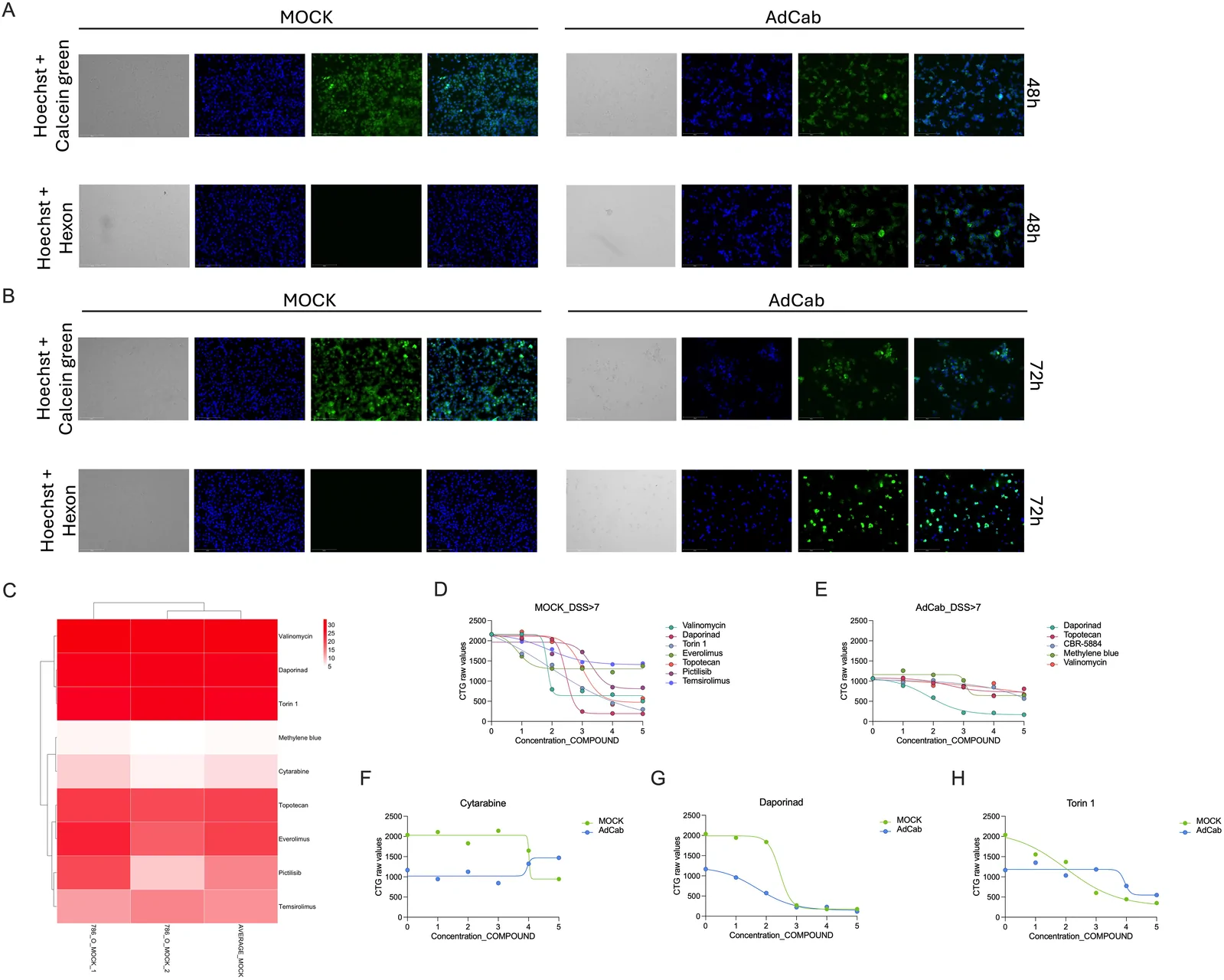
Oncolytic AdCab virus efficiently infects 786-O renal cancer cells and exhibits enhanced cytotoxicity when combined with metabolic drugs. Hexon staining confirmed successful infection of 786-O renal cancer cells with the oncolytic AdCab virus already at 48 h post infection, with increased signal at 72 h **(A, B)**. No Hexon positivity was observed in uninfected control cells at either time point. Nuclear staining (Hoechst; blue) and live cell staining (Calcein AM; green) were positive in both uninfected and infected cells at both timepoints. Brightfield images are shown for each condition and timepoint. Drug sensitivity scores for top hits in uninfected 786-O cells are shown for replicate screens alongside the average DSS **(C)**. Dose-response curves for top performing drugs in uninfected **(D)** and AdCab infected 786-O cells **(E)**. Comparison of drug sensitivity between uninfected (green) and infected (blue) cells, illustrates distinct response patterns to cytarabine **(F)**, daporinad **(G)**, and torin 1 **(H)**. Scale bars represent 275 μm.

We used the oncolytic adenovirus AdCab to demonstrate its ability to infect ccRCC cells in the 786-O cell line. Successful viral infection was assessed by monitoring expression of the adenoviral Hexon capsid protein at 48 h and 72 h timepoints. We observed a green signal originating from the green fluorescent protein (GFP) conjugated anti-hexon antibody in infected cells already at 48 h after AdCab infection (**Figure 2A**). This signal was further increased at 72 h post infection (**Figure 2B**). Cell viability was evaluated under both experimental conditions. At 48 h and 72 h post infection, viability remained positive in both uninfected and infected cells, indicating that the virus had not yet killed all cells.

Following confirmation of successful viral infection (**Figures 2A, B**), we screened 786-O cells against a selected library of 182 drugs (**Supplementary Table 2**) targeting diverse cellular metabolic pathways in both MOCK (uninfected) and AdCab (infected) conditions. Cancer cell responses to each of the compounds were evaluated using two main criteria: the drug sensitivity score (DSS) and the shape of the concentration-based response curve. Seven compounds elicited responses in 786-O MOCK cells with DSS>7 and were identified as hits (valinomycin, daporinad, torin 1, topotecan, everolimus, pictilisib and temsirolimus) (**Figure 2C**). A more detailed representation of the concentration-dependent responses of each drug is shown in the drug response curves, ranging from the negative DMSO control (x-axis value 0) to the highest tested concentration (x-axis value 5) (**Supplementary Figure 1**). In the MOCK condition (**Figure 2D**), everolimus caused a marked reduction in CTG signal, reflecting intracellular ATP levels, even at very low concentrations. Valinomycin and daporinad showed steep declines at low-to-intermediate concentrations, while topotecan and pictilisib decreased at intermediate-to-high concentrations. Contrarily, torin 1 and temsirolimus declined more gradually across the concentration range.

Building on these observations, we hypothesized that combining metabolic drugs with AdCab could further sensitize 786-O cells and enhance drug efficacy. Interestingly, applying a DSS cutoff of >7 revealed that several top hits were shared between MOCK and AdCab infected cells, including valinomycin, daporinad, and topotecan. CBR-5884 was the only drug that scored as a DSS hit exclusively in infected cells (**Figure 2E**), while everolimus and temsirolimus were identified as hits solely in uninfected cells (**Figure 2D**). While conclusions based on the mechanism of action of these drugs could not yet be drawn, we further investigated differences in drug sensitivity between infected and uninfected cells for selected shared hits. Analysis of cytarabine (**Figure 2F**), daporinad (**Figure 2G**) and torin 1 (**Figure 2H**) revealed consistent trends where uninfected cells (green line) exhibited higher viability at concentration 0 (negative control) compared to infected cells (blue line), reflecting the cytotoxic effect of AdCab infection. Beyond this shared feature, the compounds displayed distinct concentration-dependent response profiles. In the presence of daporinad (**Figure 2G**), both uninfected and infected cells reached the same viability level at 100 nanomolar (nM) (concentration 3). On the contrary, cytarabine (**Figure 2F**) and torin 1 (**Figure 2H**) both result in lower viability in the MOCK condition. Notably, torin 1 produced a progressive decrease in viability across concentrations, whereas cytarabine combined with AdCab surprisingly resulted in increased viability at the highest concentrations. Together, these findings indicate that RCC cells exhibit selective sensitivity to metabolic drug compounds both in the absence and presence of AdCab.

### Patient-derived tumor cells show drug sensitivity when assessed for combinational therapies in the presence of AdCab

**Figure 3:**
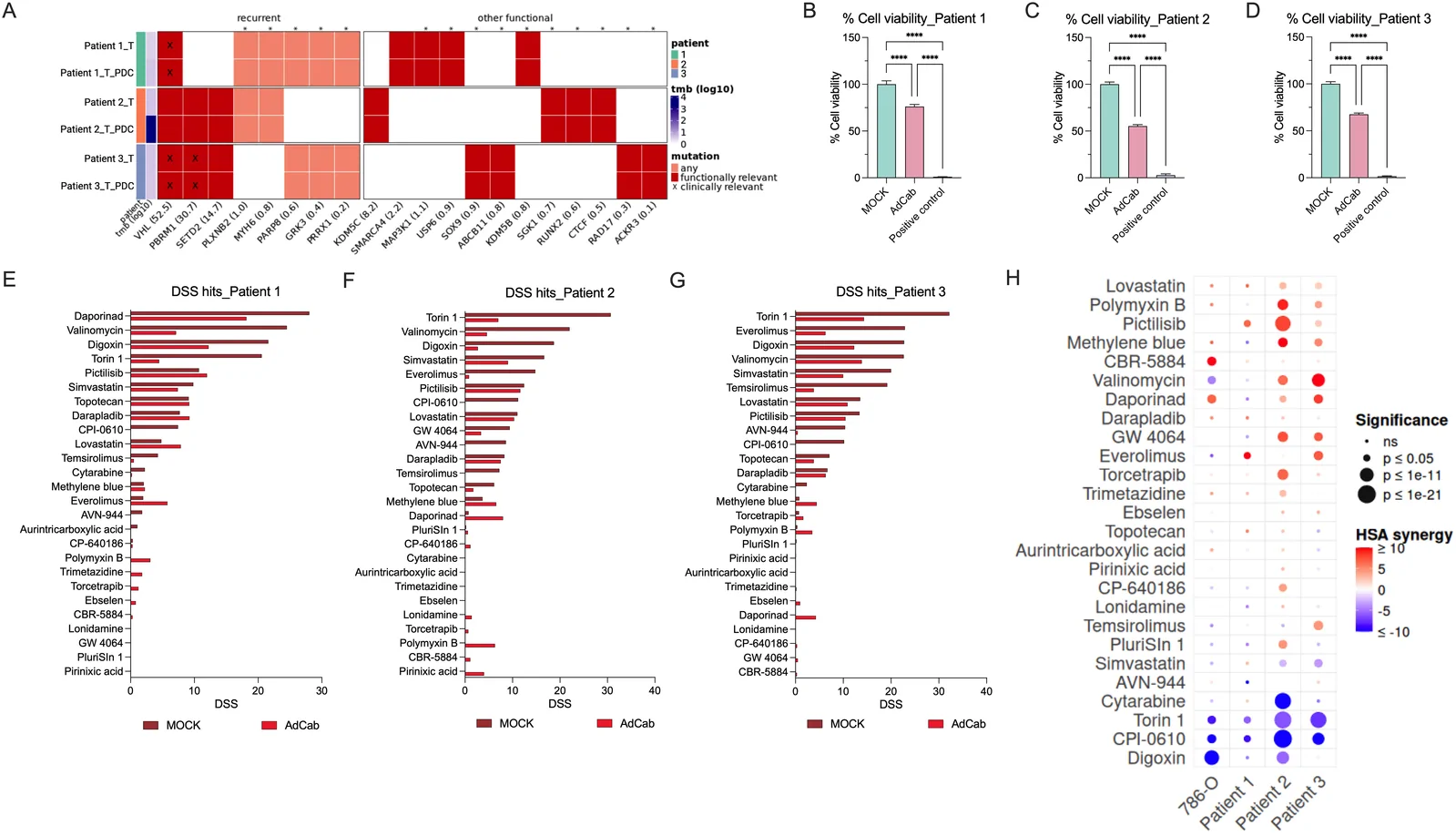
ccRCC PDCs are susceptible to AdCab infection, and the tested compounds reduce PDC viability in a dose-dependent manner, with some exhibiting synergistic activity in combination with AdCab. Mutational profiling of PDCs and corresponding parental tumor tissue demonstrated a high degree of representativeness of cultures derived from respective parental tissue **(A)**. All PDC models were susceptible to AdCab infection, resulting in reduced cell viability ranging from 50%-75% **(B–D)** after 72 h. Individual response rates for 26 selected drugs are shown for uninfected and AdCab infected PDCs from Patient 1 **(E)**, Patient 2 **(F)** and Patient 3 **(G)** with DSS values ranging from 0 to 30. Synergy analysis of AdCab in combination with 26 compounds using the SynergyFinder platform and the HSA reference framework across 786-O cells and three PDC models **(H)**. Significance levels were set at ****p < 0.0001 (two-way ANOVA).

While commercial cell lines are widely used in cancer research (33, 34), we aimed to investigate drug sensitivity using *ex vivo* PDC models, with higher clinical relevance (35, 36). Fresh tumor samples were collected during open nephrectomy from patients enrolled in the ongoing DEDUCER clinical study (19). According to the Leibovich risk group classification, three high-risk patients aged 45–65 years with grade 3–4 tumors, confirmed metastatic disease, and a TMB consistent with the TCGA reference cohort were included in this study (**Supplementary Table 1**). Tissues were processed according to previously described protocols (18) and single cell suspensions were cultured at low passages before use. To confirm that ccRCC patient-derived cells used as PDC models were representative of cancer cells of the parental tumor tissue, whole exome sequencing (WES) was performed on both tumor and matched blood samples and their corresponding PDCs. WES revealed that the clinically/functionally relevant somatic variants of the tissue that the PDCs were derived from were well preserved in PDC models (**Figure 3A**). Among the somatic mutations, VHL mutations were observed in all three patients, consistent with typical findings in RCC tumors (37–39). Patient 2 and Patient 3 also harbored two other genes commonly mutated in ccRCC, PBRM1 and SETD2 (38) (**Supplementary Table 3**).

Next, PDCs were infected with AdCab to compare the cell viability of virus-infected and uninfected cells from each patient. AdCab reduced cell viability by approximately 25-45% compared with uninfected controls across all PDCs (**Figures 3B–D**). To evaluate cell viability in the presence of drugs, we customized a compound library containing a total of 26 drug compounds (**Supplementary Table 4**) selected based on prior screens in 786-O cells (**Figure 2**). Each PDC model was screened under uninfected and AdCab infected conditions (**Figures 3E–G**). As expected, drug performance varied across the PDC models. Specifically, we identified 10/26 drugs with significantly heterogeneous response in MOCK and/or AdCab conditions (Kruskal-Wallis p ≤ 0.1 and signal-to-noise ratio (biological-to-technical SD ratio) ≥ 1.5 with median SD for MOCK: DSS 1.2 and median SD for AdCab: DSS 1.9) (**Supplementary Figures 3A–C**).

To determine whether the enhanced effects observed following combined treatment reflected true pharmacological interactions rather than additive cytotoxicity, we performed formal synergy analysis using the SynergyFinder platform (31) and the Highest Single Agent (HSA) reference model. HSA synergy scores, Relative Inhibition (RI), and Combination Sensitivity Scores (CSS) were calculated for all 26 compounds in the 786-O cell line and the three PDC models. Overall, the synergy analysis revealed compound- and patient-specific interaction profiles (**Figure 3H**). While most AdCab-drug combinations exhibited HSA scores consistent with additive effects (|HSA synergy score| ≤ 10), several compounds demonstrated positive and highly significant HSA synergy scores in specific models, indicating treatment effects exceeding those expected based on the most effective single agent alone. Notably, daporinad, together with valinomycin, everolimus, and several additional compounds, showed positive synergy scores in one or more PDC models, with the strongest synergistic interactions generally observed in Patient 2 and Patient 3, whereas combinations involving digoxin, CPI-0610, and torin 1 exhibited predominantly significantly negative HSA scores, suggesting antagonistic interactions (**Figure 3H**; **Supplementary Figure 2**). These findings demonstrate that the interaction between AdCab and targeted compounds is highly context dependent and provide quantitative pharmacological evidence that selected combinations produce synergistic effects beyond simple additivity, complementing the viability-based drug sensitivity analyses.

The robustness of the DSRT platform was supported by low variability between screening replicates (**Supplementary Figure 3A**), enabling reliable downstream analysis. Based on patient-specific (**Supplementary Figure 3D**) and concentration-dependent responses (**Supplementary Figures 3E–M**), together with activity profiles, immune-related targets, and complementary REACTOME pathway annotations (**Supplementary Table 5**), daporinad, digoxin, and everolimus were selected for combination studies with AdCab to capture distinct biological mechanisms relevant to tumor–immune interactions.

### CD8+ T-cell activation is observed upon combinational treatment with AdCab and drugs acting on immune-related pathways

**Figure 4:**
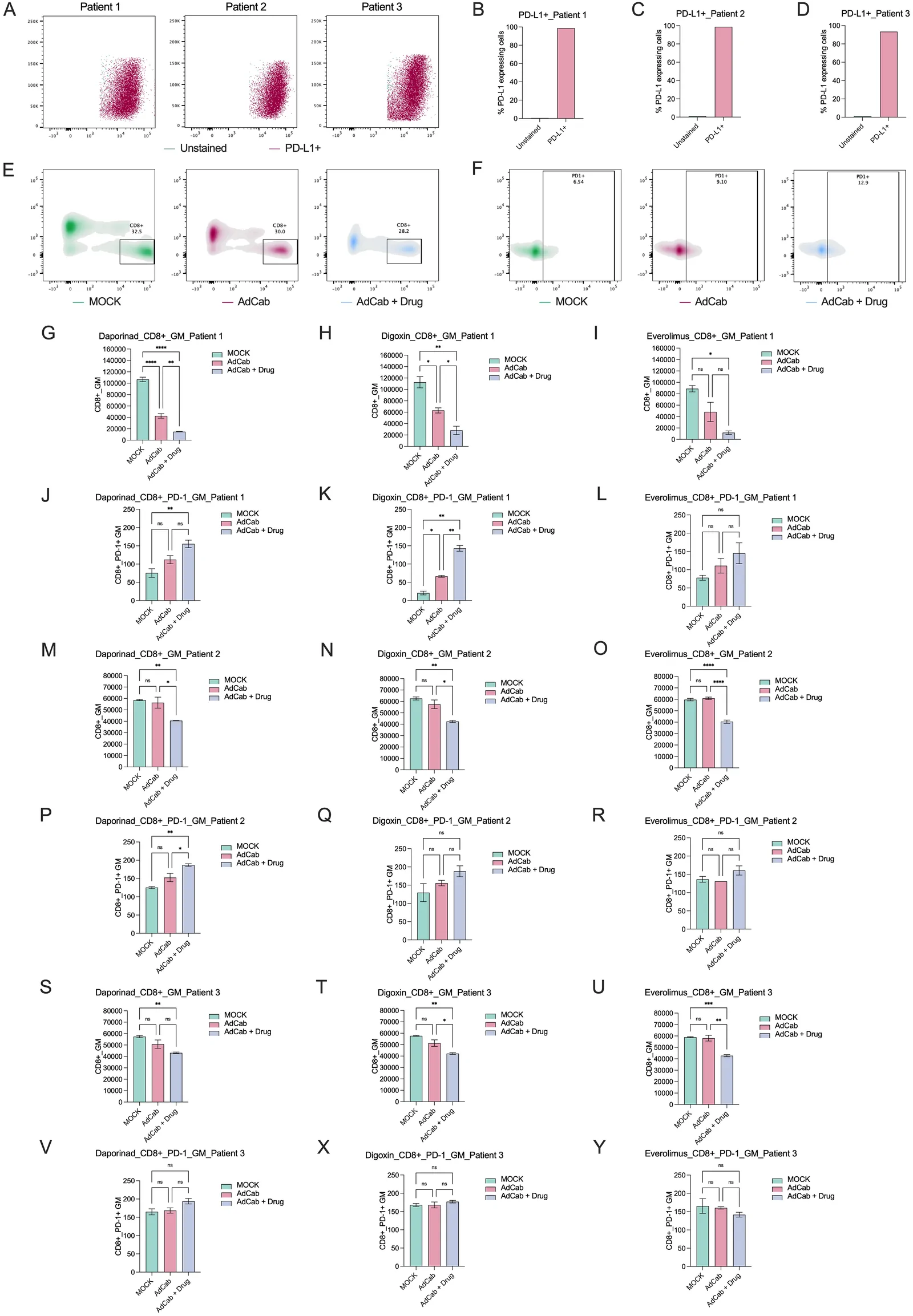
CD8+ T-cells are activated following drug and viral treatment of ccRCC PDCs. Representative flow cytometry dot plots of PD-L1 expression, including unstained controls and PD-L1 staining in ccRCC PDCs from Patients 1-3 **(A)**, with corresponding quantitative analysis **(B–D)**. CD8^+^ T-cell activation was assessed by geometric mean fluorescence intensity of CD8 and PD-1, with representative density plots for CD8 **(E)** and PD-1 **(F)** and their corresponding quantification after treatment with daporinad, digoxin, or everolimus in PDCs from Patient 1 **(G–L)**, Patient 2 **(M–R)**, and Patient 3 **(S–Y)**. Significance levels were set at *p < 0.05, **p < 0.01, ***p < 0.001 and ****p < 0.0001 (one-way ANOVA).

To confirm target availability for AdCab-mediated immune activation, PD-L1 expression was assessed in ccRCC PDCs. Representative flow cytometry dot plots of PD-L1 expression, including unstained controls and PD-L1 staining in Patients 1-3 (**Figure 4A**), are shown, followed by quantitative bar graphs depicting the proportion of PD-L1-positive cells. All PDCs exhibited high PD-L1 expression, with more than 90% of cells staining positive (**Figures 4B–D**). The proportion of PD-L1-positive cells exceeded 98% in PDCs from Patients 1 and 2 (**Figures 4B, C**), whereas PDCs from Patient 3 showed 93.4% PD-L1-positive cells (**Figure 4D**). For subsequent flow cytometry analyses, PDCs were cultured in the presence of the indicated drugs and virus, followed by a 6 h co-culture with PBMCs (**Supplementary Figures 4A, B**). To assess CD8^+^ T-cell activation, we analyzed the geometric mean (GM) of CD8 and programmed cell death protein 1 (PD-1) receptor expression. Representative density plots of CD8 (**Figure 4E**) and PD-1 (**Figure 4F**) are shown for the different treatment groups, with corresponding quantitative analyses for patients 1-3 (**Figures 4G–Y**). Effective activation is expected to correlate with a decrease in CD8 expression and a parallel increase in PD-1 expression (40, 41). Consistent with the quantitative analyses, PDCs from Patients 1 and 2 showed increased overall immune activation, particularly in CD8^+^ T cells (**Figures 4G–Y**). A clear reduction of the CD8 receptor was observed in AdCab treated Patient 1 cells, which was further enhanced when AdCab was combined with drug compounds, supporting successful T-cell activation in a combinational therapy approach (**Figures 4G–I**). Stronger decrease in CD8 expression in Patient 1 vs Patient 2 and Patient 3 PDCs was observed for digoxin, everolimus and, most notably, for daporinad (**Figures 4G, M, S**). In parallel, PD-1 expression increased most significantly in the presence of daporinad and digoxin compared to everolimus (**Figures 4J–L**) in Patient 1. Patient 2 and Patient 3 PDCs showed the same trend, with a decrease in CD8 expression (**Figures 4M–O**, **S–U**) and an increase in PD-1 expression (**Figures 4P–R**, **V–Y**), although these differences across the experimental conditions were less prominent than those observed in Patient 1 PDCs.

### NK cell recruitment and activation is induced by viral infection and drug treatment in ccRCC PDCs

**Figure 5:**
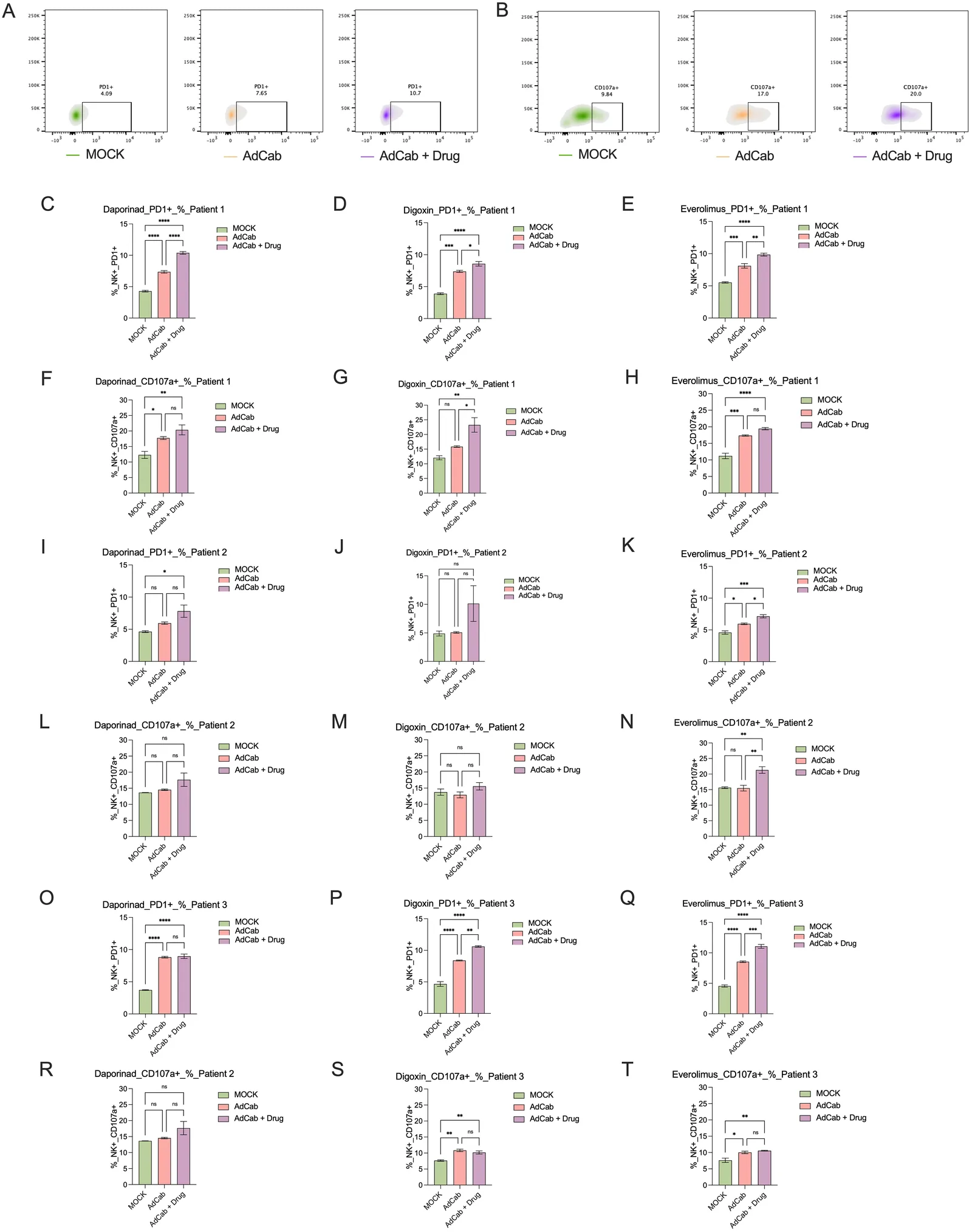
Recruitment and activation of PD-1^+^ NK cells is increased in drug and viral treated ccRCC PDCs. Representative density plots of PD-1^+^ **(A)** and CD107a^+^ **(B)** NK cells following MOCK, AdCab, and AdCab plus drug treatment. Corresponding quantitative analyses of the proportion of PD-1^+^ and CD107a^+^ NK cells following treatment with AdCab alone or in combination with daporinad, digoxin, or everolimus are shown for PDCs from Patient 1 **(C–H)**, Patient 2 **(I–N)**, and Patient 3 **(O–T)**. Significance levels were set at *p < 0.05, **p < 0.01, ***p < 0.001 and ****p < 0.0001 (one-way ANOVA).

After observing increased CD8^+^ T-cell activation across all ccRCC PDC models, and given that AdCab, due to its IgG1 hybrid backbone, has previously been shown to stimulate NK cell-mediated responses in mice (25), we extended our analysis to evaluate the proportion of PD-1^+^ NK cells in human ccRCC PDC models. Using the same experimental workflow as for CD8^+^ T-cell analyses, purified NK cells were used as effector cells, and the proportion of both PD-1^+^ and CD107a^+^ NK cells was determined by flow cytometry (**Supplementary Figures 5A, B**). Representative density plots of PD-1^+^ (**Figure 5A**) and CD107a^+^ (**Figure 5B**) NK cells following MOCK, AdCab, and AdCab plus drug treatment are shown. A similar trend to that observed with PBMCs as effector cells was also seen using purified NK cells. The combination of AdCab with each of the selected drug compounds (daporinad, digoxin, or everolimus) consistently increased the proportion of PD-1^+^ NK cells across all PDC models; Patient 1 (**Figures 5C–E**), Patient 2 (**Figures 5I–K**), and Patient 3 (**Figures 5O–Q**). Notably, AdCab alone also increased PD-1^+^ NK cell frequencies compared with MOCK, while the combination treatment (AdCab plus drug) consistently showed the strongest effect across all models and compounds. NK cell activation was further assessed using CD107a expression, a marker of degranulation and cytotoxicity. AdCab alone and in combination with drugs generally increased CD107a^+^ NK cell frequencies compared with MOCK across all PDC models (Patient 1, **Figures 5F–H**; Patient 2, **Figures 5L–N**; Patient 3, **Figures 5R–T**). An exception was observed in Patient 2 PDCs treated with daporinad (**Figure 5L**) and everolimus (**Figure 5N**), where MOCK exceeded AdCab alone; however, AdCab plus drug combination treatment still resulted in higher CD107a^+^ NK cell frequencies compared to MOCK.

### Daporinad induces a reproducible spatial remodeling phenotype across PDC co-cultures

**Figure 6:**
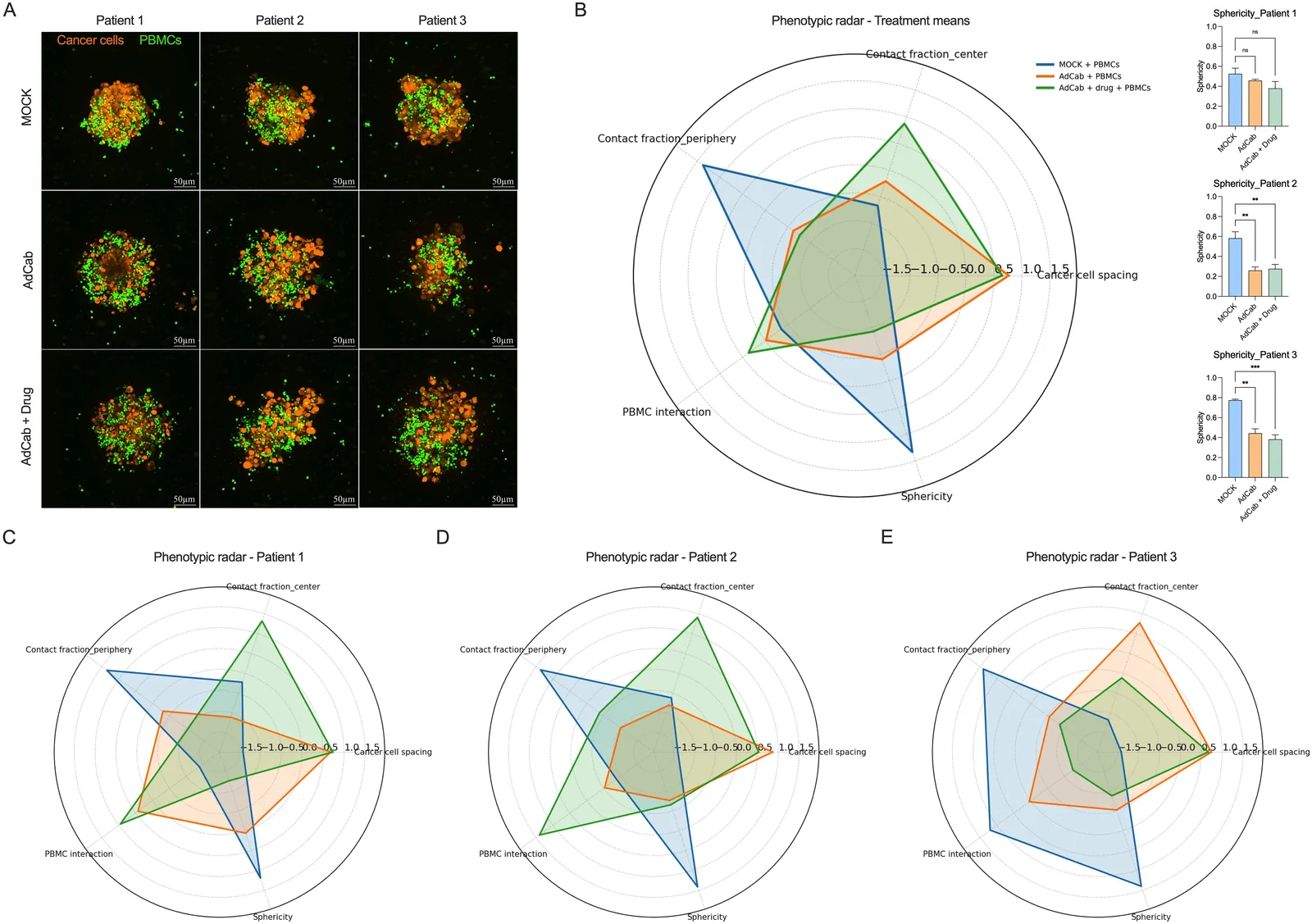
Daporinad treatment is associated with a spatial remodeling phenotype in PDC co-cultures. Spheroids of Patient 1, Patient 2 and Patient 3 PDCs (orange), uninfected, infected with AdCab or treated with the combination of AdCab and daporinad in the presence of human PBMCs (green) **(A)**. Radar profile means of three patients´ PDCs **(B)**, with an example of single responses (1 = perfect sphere; 0 = irregular or flattened) representing the sphericity feature mean across all PDCs, reveal a highly reproducible directional response to daporinad treatment. Patient 1, Patient 2 and Patient 3 PDCs each retain subtle, biologically meaningful nuances in the amplitude and feature weighting of this response **(C–E)**. Scale bars represent 50µm. Significance levels were set at **p < 0.01 and ***p < 0.001 (one-way ANOVA).

To assess the morphological effect of drugs and virus on PDCs in co-culture with human immune cells, we performed imaging and quantitative image analysis on daporinad-treated cultures given its previously demonstrated ability to induce strong CD8+ T-cell activation across all PDC models (**Figure 4**). Spheroids were acquired using the Opera Phenix high-content microscopy system under confocal mode and processed using an in-house developed analysis framework (32). This pipeline combines deep learning-based segmentation and Bayesian optimization to achieve single-cell resolution within each 3D spheroid. From the segmented data, multiscale morphological features were extracted, including spheroid-level compactness, cell spacing, and surface sphericity, as well as local cellular density and contact metrics, enabling direct quantification of treatment-induced architectural remodeling. Consistent with cell viability data obtained from the AdCab infection susceptibility assessment (**Figures 3B–D**), we observed increased spheroid dissociation upon AdCab treatment compared with uninfected controls, an effect typically associated with advanced viral infection and cell death. This structural dissociation was further enhanced in all PDC models when tumor cells were treated with the combination of AdCab and daporinad (**Figure 6A**). Alongside visual assessment of spheroid morphology, we quantified cancer cell scattering based on nearest-neighbor distances (NNDs). Spatial dispersion of cancer cells within spheroids reflected by this metric was expressed as the mean NND normalized to the sphere radius. In all PDC models (Patient 1-3), AdCab treatment increased the NND values (**Supplementary Figures 6A–C**) indicating enhanced spheroid dissociation following viral infection. To further investigate cross cell type interactions, we performed regional analyses comparing the spheroid center and its periphery to detect cell localization and engagement across treatment conditions. The contact fraction, defined as the proportion of cells possessing at least one opposite-type neighbor within a fixed distance, was highest in the spheroid centers of Patient 1 and Patient 2 compared to Patient 3 (**Supplementary Figures 6D–F**) after treatment with AdCab and daporinad. In contrast, Patient 3 showed the highest central contact fraction after AdCab treatment alone (**Supplementary Figure 6F**). At the sphere periphery, contact fractions were the highest for MOCK conditions and lowest following AdCab or AdCab plus daporinad treatment across all PDCs (**Supplementary Figures 6G–I**), supporting earlier sphericity measurements (**Figure 6B**). To further determine a potential advantage of combination treatment with AdCab and daporinad, we assessed interactions between cancer cells and PBMCs within the co-cultures. For PDCs from Patient 1 and Patient 2 (**Supplementary Figures 6J, K**), AdCab plus daporinad induced higher proportions of interactions, reflecting increased spatial mixing of cancer and immune cells compared to AdCab alone or MOCK. In contrast, no such increase was observed for Patient 3 (**Supplementary Figure 6L**).

Building on the single-feature quantifications, we next integrated these morphological descriptors into a multivariate phenotypic framework to further characterize the observed trends (**Figure 6**; **Supplementary Figure 6**). Daporinad was associated with decreased spheroid sphericity and peripheral contact fraction, together with increased cancer cell spacing across the PDC co-cultures. The averaged radar plot profile revealed a similar directional phenotypic pattern across patient models, where both AdCab and AdCab plus daporinad-treated spheroids exhibited reduced sphericity and peripheral contact fraction alongside increased cancer cell spacing, reflecting a shift from compact, spherical morphologies toward more open and irregular architectures (**Figure 6B**). Individual sphericity values for each patient are shown together with the mean sphericity in the radar plot (**Figure 6B**), indicating a reduction following AdCab and combination treatment compared with MOCK controls. When Patient 1 (**Figure 6C**), Patient 2 (**Figure 6D**), and Patient 3 (**Figure 6E**) models were analyzed individually, the patient-specific radar profiles showed comparable directional changes, suggesting that these morphometric trends were not driven by a single model but reflected consistent phenotypic responses across the included PDCs. Although direct quantification of single-cell interactions remains challenging in this system, these results highlight the utility of high-content image analysis for capturing treatment-associated spatial phenotypic changes in ccRCC models.

## Discussion

In this study, the therapeutic efficacy of metabolic compounds and oncolytic viruses on a ccRCC cell line and three clinically representative PDCs from patients with high-risk ccRCC was evaluated. An initial high-throughput DSRT screen with 182 metabolic compounds in 786-O cells identified 26 drug candidates with cytotoxic activity, which were subsequently profiled in all three PDC models to assess combinational effects with the oncolytic AdCab virus. Statistical analysis across all models prioritized three top-performing drugs; daporinad, digoxin and everolimus, for deeper investigation of their immunomodulatory synergy in tumor cell elimination. We showed that AdCab efficiently infected ccRCC target cells and induced cancer cell killing as a monotherapy. This effect was further enhanced when combined with CD8+ T-cells and NK cells, demonstrating a synergistic antitumor response. This effect was further potentiated by the addition of daporinad, digoxin and everolimus, thereby offering a novel therapeutic strategy for treating ccRCC. Quantitative image analysis finally revealed that daporinad treatment induced spatial remodeling in all PDC co-cultures. These findings provide a rational combination strategy leveraging metabolic modulation and oncolytic virotherapy to enhance antitumor immunity, providing a mechanistic foundation for the development of improved immuno-oncology interventions in RCC.

The standard treatment for RCC involves partial nephrectomy or surgical resection, in which the cancerous tissue and a small margin of surrounding kidney are removed. This approach is often curative in patients with low-stage tumors and benefits patients by preserving as much renal tissue as possible (42, 43). For patients who do not benefit from standard treatment or are not candidates for surgical resection, additional treatment options include adjuvant therapy, immunotherapy or targeted therapy. In this study, it is demonstrated that AdCab markedly reduced cell viability in the ccRCC cell line and PDCs. Oncolytic adenovirus therapies have previously demonstrated considerable promise in cancer treatment, owing to the heightened susceptibility of tumor cells to virus-mediated killing (44–47), though short-term experiments often leave a residual population of cancer cells unaffected. In clinical practice, combining oncolytic virus treatment with pharmacological agents – either to enhance viral infectivity or to act synergistically – may improve cancer cell eradication. In our study, a synergistic effect was observed with AdCab in combination with daporinad, an NAMPT inhibitor. Daporinad depletes intracellular NAD+ levels, enhancing cytotoxicity through signaling pathways involving e.g. SIRT1 and p53 (48). The observed effect may involve different mechanisms including the sensitization of cancer cells to viral infection by the NAMPT inhibitor, and/or the enhanced delivery of daporinad to target cells mediated by the virus, to potentially overcome drug resistance (49, 50). Interestingly, combining NAMPT inhibitors with anti-PD-1 has been shown to shift macrophage polarization toward the immunosuppressive M2 phenotype (51) while also increasing T-cell recruitment and prolonging survival of *in vivo* mouse models (52). In RCC, NAMPT inhibitors have been reported to reduce invasion and migration, decrease cell viability, and induce apoptosis (50). However, these observations are primarily based on the drug mechanism and require further experimental validation and careful evaluation.

In our study, we observed that treatment with digoxin, a clinically approved cardiac glycoside used for heart failure and specific arrhythmias, led to reduced tumor cell viability and increased T-cell activation in all PDCs. While digoxin remains in the investigational phase for cancer treatment, studies suggest that it can inhibit tumor cell proliferation through blockade of the Na+/K+ ATPase pump (53–55). Interestingly, combining digoxin with TKIs has been suggested to potentially improve survival outcomes in RCC patients (56). Consistent with this observation, both digoxin and daporinad achieved higher DSS compared to everolimus in AdCab treated PDCs from Patient 1 and Patient 2 (**Figures 3E, F** and **Supplementary Figure 3D**). This finding suggests increased drug sensitivity and reduced viability of PDCs following virus-drug combination treatment.

CD8+ T cells serve as pivotal effectors in the immune response against tumors, contributing to immune surveillance. Modeling of interactions between T cells and cancer cells revealed patient-specific variability in drug efficacy. In Patient 1 PDCs, both digoxin and daporinad induced stronger overall CD8^+^ T-cell activation compared to everolimus (**Figures 4G–L**). A comprehensive comparison of all three drug compounds and CD8^+^ T-cell activation across all PDC models revealed that Patient 1 treated with daporinad consistently exhibited the strongest response, outperforming all other experimental combinations based on statistical significance (one-way ANOVA) (**Figures 4G–Y**). On the contrary, everolimus treatment was associated with higher CD8+ T-cell GM in Patient 2 (**Figures 4M–O**) and Patient 3 (**Figures 4S–U**) compared to Patient 1. Given the established use of the mTOR inhibitor everolimus in treating ccRCC patients, particularly after failure of first-line VEGFR treatment (57, 58), and its ability to augment the efficacy of oncolytic adenoviruses (59), this drug was considered a positive control in our study.

While patient-specific differences likely contribute to variations in drug response, factors like PBMC composition and the activation status of CD8+ T-cells, reflecting the coordinated biological function of immune and target cells, are also likely to be involved. An increased percentage of PD-1^+^ NK cells was observed when PDCs were treated with AdCab, with an even greater increase in combination with drugs (**Figures 5C–E, I–K, O–Q**). This pattern was consistent across all PDC models and drug compounds tested. As central effectors of antitumor immunity and key targets of immune checkpoint pathways, NK cells and CD8^+^ T cells were both elevated following combination treatment, indicating that AdCab plus drugs can potentiate immune responses (60). CD107a was employed as a functional indicator of NK cell degranulation, reflecting Fc receptor (CD16A)-mediated activation during antibody-dependent cell-mediated cytotoxicity (ADCC). This assay captures effector function driven by Fc-CD16A engagement rather than antigen-specific recognition. By contrast, CD107a mobilization and intracellular cytokine staining are most informative in CD8^+^ T cells under conditions of defined antigen stimulation and TCR engagement and are therefore not directly interpretable in an unsensitized PBMC-tumor co-culture system (61). In this setting, PD-1 was used as an early activation-associated marker for CD8^+^ T cells. PD-1 is rapidly induced following acute stimulation and can reflect transient activation in short-term assays, whereas sustained expression is typically associated with chronic antigen exposure and functional exhaustion (62). Accordingly, CD107a and PD-1 readouts should be interpreted in a context-dependent manner, representing Fc/CD16A-driven NK effector activity and acute CD8^+^ T-cell activation, respectively. Mechanistically, PD-1 engagement with its ligands, primarily PD-L1 on tumor cells, is known to suppress NK cell cytotoxicity (63, 64). Although the impact of PD-1^+^ NK cells on tumor immunity remains controversial (65–67), PD-1/PD-L1 blockade is generally suggested as an efficient approach for increasing NK cell activity. However, its efficacy likely depends on tumor type, the TME, and the expression levels of relevant surface markers, underscoring the need for detailed, context-specific evaluation in future studies.

Building on the observed effects of combining AdCab with drugs, particularly daporinad, we investigated cancer cell spheroid and PBMC interactions using phenotypic microscopy-based imaging. Although light penetration into spheroids introduced some analytical limitations, the results were consistent with our previous observations showing increased immune cell engagement with cancer cells following combined treatment in two out of three PDC models (**Figure 6**; **Supplementary Figure 6**). Further analysis of spatial remodeling phenotypes in the PDC models revealed that Patient 1 PDCs exhibited the most pronounced differences between MOCK, AdCab, and AdCab plus daporinad conditions (**Figure 6C**). The MOCK condition showed a compact morphology characterized by high sphericity and strong peripheral contact fraction, consistent with densely organized spheroids. In contrast, both AdCab and AdCab plus daporinad were associated with reduced sphericity and peripheral contact, together with increased intercellular spacing, reflecting a loosening of spheroid architecture. The combined treatment condition additionally showed increased PBMC proximity and central contact fractions compared with single-agent treatment. These observations are consistent with enhanced immune cell infiltration or redistribution within the spheroid structure under combination treatment conditions, although definitive mechanistic attribution cannot be established from imaging data alone. Collectively, these findings support an association between combination treatment and altered tumor-immune spatial organization in ccRCC spheroid models, highlighting the potential of integrating pharmacological agents with immunotherapy approaches for further investigation.

When using oncolytic virus therapies, it is essential to note that some compounds may suppress viral infection. Conversely, the virus may also impact the effectiveness of certain drugs by altering cellular signaling pathways involved in their mechanism of action. In our study, uninfected cancer cells were highly sensitive to the mTOR inhibitor torin 1, while the presence of AdCab clearly reduced their sensitivity (**Figure 2H**). More strikingly, cytarabine, a chemotherapeutic antimetabolite, significantly reduced viability in uninfected cells, while AdCab infected cells continued to proliferate despite drug exposure (**Figure 2F**). As cytarabine targets rapidly dividing cells in the S-phase of the cell cycle, AdCab infection may potentially arrest cells in a different phase, which consequently would reduce drug efficacy.

While everolimus has been widely used in the clinic, digoxin and daporinad are not yet part of standard care for ccRCC, highlighting opportunities for novel therapeutic strategies to improve patient outcomes. Although drug treatment alone can achieve tumor-free survival in some patients, the human immune system plays a critical role in determining treatment response. In this context, we show that AdCab effectively enhances tumor cell killing when combined with drugs targeting immune-related pathways and activates immune cell populations, particularly cytotoxic CD8+ T cells, thereby promoting more effective tumor regression. We note that these findings are based on a limited number of PDC models and should therefore be interpreted as preliminary, warranting validation in larger cohorts to confirm their generalizability. While our PDC-based co-culture system incorporating immune effector cells provides a physiologically relevant platform for assessing therapeutic responses, *in vivo* studies will be required to evaluate antitumor efficacy, immune modulation, and safety in a systemic context. Despite these limitations, our findings provide insights into ccRCC combination therapies that achieve effective tumor cell killing in the presence of both drug compounds and human immune cells. Overall, our results indicate that digoxin and daporinad represent promising drug candidates for ccRCC therapy that warrant further safety evaluation in clinical trials and future translational investigation.

## Data Availability

The datasets presented in this article are not readily available because, according to our ethical statement, we cannot publish this data fully due to personal information enclosed (i.e. could be identifiable if all genetic data in the public repository). Requests to access the datasets should be directed to the corresponding author(s).

## References

[1] Wu X Lazris D Wong R Tykodi SS . Belzutifan for the treatment of renal cell carcinoma. Ther Adv Med Oncol. (2025) 17:17588359251317846. doi: 10.1177/17588359251317846 39926260 PMC11806488

[2] Chen AH Grana AK . Belzutifan’s role in the treatment landscape of clear cell renal cell carcinoma. Pharmacotherapy. (2025) 45:356–66. doi: 10.1007/s11864-023-01161-5 40331637

[3] Kaelin WG . The VHL tumor suppressor gene: Insights into oxygen sensing and cancer. Trans Am Clin Climatol Assoc. (2017) 128:298. doi: 10.1016/s0959-437x(02)00010-2 28790514 PMC5525432

[4] Fiorentino V Tralongo P Larocca LM Pizzimenti C Martini M Pierconti F . First-line ICIs in renal cell carcinoma. Hum Vaccin Immunother. (2023) 19(2):2225386. doi: 10.1080/21645515.2023.2225386 37395601 PMC10332208

[5] Chen X Ke Jianan CY Chen Z Ke Y Huang X . Immunotherapy in clear cell renal cell carcinoma: current status, novel strategies, and future perspectives. Clin Exp Med. (2026) 26:116. doi: 10.1007/s10238-025-02031-0 41543784 PMC12847155

[6] Lopez-Beltran A Henriques V Cimadamore A Santoni M Cheng L Gevaert T . The identification of immunological biomarkers in kidney cancers. Front Oncol. (2018) 8:456. doi: 10.3389/fonc.2018.00456 30450335 PMC6225533

[7] Steiner C Machaalani M Bonventre JV McDermott DF Choueiri TK Xu W . KIM-1 as a prognostic marker in renal cell carcinoma. Eur Urol Focus. (2025) 11:432–5. doi: 10.1016/j.euf.2025.06.012 40619332 PMC13478781

[8] Wang X Zhong L Zhao Y . Oncolytic adenovirus: A tool for reversing the tumor microenvironment and promoting cancer treatment (review). In: Oncol. Rep (2021). Available online at: https://www.spandidos-publications.com/10.3892/or.2021.8000 (Accessed July 10, 2025). 10.3892/or.2021.8000 PMC7934214 33760203

[9] Khalaf K Hana D Chou JTT Singh C Mackiewicz A Kaczmarek M . Aspects of the tumor microenvironment involved in immune resistance and drug resistance. Front Immunol. (2021) 12:656364. doi: 10.3389/fimmu.2021.656364 34122412 PMC8190405

[10] Sun Y . Tumor microenvironment and cancer therapy resistance. Cancer Lett. (2016) 380:205–15. doi: 10.1016/j.canlet.2015.07.044 26272180

[11] Sato-Dahlman M Larocca CJ Yanagiba C Yamamoto M . Adenovirus and immunotherapy: Advancing cancer treatment by combination. Cancers. (2020) 12:1295. doi: 10.3390/cancers12051295 32455560 PMC7281656

[12] Dias JD Hemminki O Diaconu I Hirvinen M Bonetti A Guse K . Targeted cancer immunotherapy with oncolytic adenovirus coding for a fully human monoclonal antibody specific for CTLA-4. Gene Ther. (2012) 19:988–98. doi: 10.1038/gt.2011.176 22071969

[13] Block MS Clubb JHA Mäenpää J Pakola S Quixabeira DCA Kudling T . The oncolytic adenovirus TILT-123 with pembrolizumab in platinum resistant or refractory ovarian cancer: the phase 1a PROTA trial. Nat Commun. (2025) 16:1–14. doi: 10.1038/s41467-025-56482-w 39910037 PMC11799410

[14] Ponce S Cedrés S Ricordel C Isambert N Viteri S Herrera-Juarez M . ONCOS-102 plus pemetrexed and platinum chemotherapy in Malignant pleural mesothelioma: a randomized phase 2 study investigating clinical outcomes and the tumor microenvironment. J Immunother Cancer. (2023) 11:7552. doi: 10.1136/jitc-2023-007552 37661097 PMC10476122

[15] Musher BL Rowinsky EK Smaglo BG Abidi W Othman M Patel K . LOAd703, an oncolytic virus-based immunostimulatory gene therapy, combined with chemotherapy for unresectable or metastatic pancreatic cancer (LOKON001): results from arm 1 of a non-randomised, single-centre, phase 1/2 study. Lancet Oncol. (2024) 25:488–500. doi: 10.1016/s1470-2045(24)00079-2 38547893 PMC12342118

[16] Jiang N Zheng Y Ding J Wang J Zhu F Wang M . The co-delivery of adenovirus-based immune checkpoint vaccine elicits a potent anti-tumor effect in renal carcinoma. NPJ Vaccines. (2023) 8:1–13. doi: 10.1038/s41541-023-00706-x 37542081 PMC10403580

[17] Viel S Vivier E Walzer T Marçais A . Targeting metabolic dysfunction of CD8 T cells and natural killer cells in cancer. Nat Rev Drug Discov. (2024) 24:190–208. doi: 10.1038/s41573-024-01098-w 39668206

[18] Feodoroff M Mikkonen P Arjama M Murumägi A Kallioniemi O Potdar S . Protocol for 3D drug sensitivity and resistance testing of patient-derived cancer cells in 384-well plates. SLAS Discov. (2023) 28:36–41. doi: 10.1016/j.slasd.2022.11.003 36464160

[19] Feodoroff M Luck TJ Kumari R Polso M Penttilä P Malmstedt M . Site-dependent decoupling of drug-biomarker associations in clear cell renal cell carcinoma revealed by functional profiling of patient-derived cell models. bioRxiv. (2026), 2026.04.23.720088. doi: 10.64898/2026.04.23.720088

[20] Saeed K Ojamies P Pellinen T Eldfors S Turkki R Lundin J . Clonal heterogeneity influences drug responsiveness in renal cancer assessed by ex vivo drug testing of multiple patient-derived cancer cells. Int J Cancer. (2019) 144:1356–66. doi: 10.1002/ijc.31815 30125350

[21] Wang K Li M Hakonarson H . ANNOVAR: functional annotation of genetic variants from high-throughput sequencing data. Nucleic Acids Res. (2010) 38:e164–e164. doi: 10.1093/nar/gkq603 20601685 PMC2938201

[22] Li Q Wang K . InterVar: Clinical interpretation of genetic variants by the 2015 ACMG-AMP guidelines. Am J Hum Genet. (2017) 100:267–80. doi: 10.1016/j.ajhg.2017.01.004 28132688 PMC5294755

[23] Nakken S Fournous G Vodák D Aasheim LB Myklebost O Hovig E . Personal cancer genome reporter: variant interpretation report for precision oncology. Bioinformatics. (2018) 34:1778–80. doi: 10.1093/bioinformatics/btx817 29272339 PMC5946881

[24] Hamdan F Martins B Feodoroff M Giannoula Y Feola S Fusciello M . GAMER-Ad: a novel and rapid method for generating recombinant adenoviruses. Mol Ther Methods Clin Dev. (2021) 20:625–34. doi: 10.1016/j.omtm.2021.01.014 33718513 PMC7907680

[25] Hamdan F Ylösmäki E Chiaro J Giannoula Y Long M Fusciello M . Novel oncolytic adenovirus expressing enhanced cross-hybrid IgGA Fc PD-L1 inhibitor activates multiple immune effector populations leading to enhanced tumor killing *in vitro*, *in vivo* and with patient-derived tumor organoids. J Immunother Cancer. (2021) 9:3000. doi: 10.1136/jitc-2021-003000 34362830 PMC8351494

[26] Potdar S Ianevski A Mpindi JP Bychkov D Fiere C Ianevski P . Breeze: an integrated quality control and data analysis application for high-throughput drug screening. Bioinformatics. (2020) 36:3602–4. doi: 10.1093/bioinformatics/btaa138 32119072 PMC7267830

[27] Zhou Y Zhang Y Zhao D Yu X Shen X Zhou Y . TTD: Therapeutic target database describing target druggability information. Nucleic Acids Res. (2024) 52:D1465–77. doi: 10.1093/nar/gkad751 PMC10767903 37713619

[28] Liberzon A Birger C Thorvaldsdóttir H Ghandi M Mesirov JP Tamayo P . The molecular signatures database (MSigDB) hallmark gene set collection. Cell Syst. (2015) 1:417. doi: 10.1016/j.cels.2015.12.004 26771021 PMC4707969

[29] Milacic M Beavers D Conley P Gong C Gillespie M Griss J . The Reactome pathway knowledgebase 2024. Nucleic Acids Res. (2024) 52:D672–8. doi: 10.1093/nar/gkad1025 37941124 PMC10767911

[30] Subramanian A Tamayo P Mootha VK Mukherjee S Ebert BL Gillette MA . Gene set enrichment analysis: A knowledge-based approach for interpreting genome-wide expression profiles. Proc Natl Acad Sci USA. (2005) 102:15545–50. doi: 10.1073/pnas.0506580102 16199517 PMC1239896

[31] Zheng S Wang W Aldahdooh J Malyutina A Shadbahr T Tanoli Z . SynergyFinder Plus: Toward better interpretation and annotation of drug combination screening datasets. Genomics Proteomics Bioinf. (2022) 20:587–96. doi: 10.1016/j.gpb.2022.01.004 35085776 PMC9801064

[32] Isabel Mogollon A Feodoroff M Neto P Montedeoca A Pietiänen V Paavolainen L . Achieving high-resolution single-cell segmentation in convoluted cancer spheroids via Bayesian optimization and deep-learning. In: Biorxiv (2024). p. 2024.09.08.611898.

[33] Gillet JP Varma S Gottesman MM . The clinical relevance of cancer cell lines. JNCI J Natl Cancer Institute. (2013) 105:452. doi: 10.1093/jnci/djt007 23434901 PMC3691946

[34] Masters JRW . Human cancer cell lines: Fact and fantasy. Nat Rev Mol Cell Biol. (2000) 1:233–6. doi: 10.1038/35043102 11252900

[35] Qu S Xu R Yi G Li Z Zhang H Qi S . Patient-derived organoids in human cancer: a platform for fundamental research and precision medicine. Mol BioMed. (2024) 5:6. doi: 10.1186/s43556-023-00165-9 38342791 PMC10859360

[36] Piwocka O Suchorska WM Kulcenty K . Empowering personalized medicine: unleashing the potential of patient-derived explants in clinical practice. EXCLI J. (2024) 23:81. doi: 10.17179/excli2023-6700 38343742 PMC10853634

[37] van de Pol JAA Ferronika P Westers H van Engeland M Terpstra MM Smits KM . Evaluation of a seven gene mutational profile as a prognostic factor in a population-based study of clear cell renal cell carcinoma. Sci Rep. (2022) 12:1–12. doi: 10.1038/s41598-022-10455-x 35444164 PMC9021193

[38] Rizzo M Pezzicoli G Porta C Povero M Pradelli L Sicari E . The genomic landscape of metastatic clear-cell renal cell carcinoma and its prognostic value: a comprehensive analysis of a large real-world clinico-genomic database. ESMO Open. (2025) 10:104294. doi: 10.1016/j.esmoop.2025.104294 39965361 PMC11876921

[39] Xie D Li G Zheng Z Zhang X Wang S Jiang B . The molecular code of kidney cancer: a path of discovery for gene mutation and precision therapy. Mol Aspects Med. (2025) 101:101335. doi: 10.1016/j.mam.2024.101335 39746268

[40] Liu Y Liang X Dong W Fang Y Lv J Zhang T . Tumor-repopulating cells induce PD-1 expression in CD8+ T cells by transferring kynurenine and AhR activation. Cancer Cell. (2018) 33:480–494.e7. doi: 10.1016/j.ccell.2018.02.005 29533786

[41] Moseman JE Rastogi I Jeon D McNeel DG . PD-1 blockade employed at the time CD8+ T cells are activated enhances their antitumor efficacy. J Immunother Cancer. (2025) 13:11145. doi: 10.1136/jitc-2024-011145 40341032 PMC12067779

[42] Nguyen CT Campbell SC Novick AC . Choice of operation for clinically localized renal tumor. Urologic Clinics North America. (2008) 35:645–55. doi: 10.1016/j.ucl.2008.07.002 18992618

[43] Chung JS Son NH Lee SE Hong SK Lee SC Kwak C . Overall survival and renal function after partial and radical nephrectomy among older patients with localised renal cell carcinoma: a propensity-matched multicentre study. Eur J Cancer. (2015) 51:489–97. doi: 10.1016/j.ejca.2014.12.012 25576517

[44] Lin D Shen Y Liang T . Oncolytic virotherapy: basic principles, recent advances and future directions. Signal Transduction Targeted Ther. (2023) 8:1–29. doi: 10.1038/s41392-023-01407-6 37041165 PMC10090134

[45] Peter M Kühnel F . Oncolytic adenovirus in cancer immunotherapy. Cancers (Basel). (2020) 12:3354. doi: 10.3390/cancers12113354 33202717 PMC7697649

[46] Nadafi R Dong W van Beusechem VW . Immunological impact of oncolytic adenoviruses on cancer therapy: clinical insights. Eur J Immunol. (2025) 55:e70024. doi: 10.1002/eji.70024 40726054 PMC12304604

[47] Russell SJ Peng KW Bell JC . Oncolytic virotherapy. Nat Biotechnol. (2012) 30:658. doi: 10.1038/nbt.2287 22781695 PMC3888062

[48] Kennedy BE Sharif T Martell E Dai C Kim Y Lee PWK . NAD+ salvage pathway in cancer metabolism and therapy. Pharmacol Res. (2016) 114:274–83. doi: 10.1016/j.phrs.2016.10.027 27816507

[49] Ozgencil F Gunindi HB Eren G . Dual-targeted NAMPT inhibitors as a progressive strategy for cancer therapy. Bioorg Chem. (2024) 149:107509. doi: 10.1016/j.bioorg.2024.107509 38824699

[50] Aboud OA Chen CH Senapedis W Baloglu E Argueta C Weiss RH . Dual and specific inhibition of NAMPT and PAK4 by KPT-9274 decreases kidney cancer growth. Mol Cancer Ther. (2016) 15:2119–29. doi: 10.1158/1535-7163.mct-16-0197 27390344 PMC5010932

[51] Audrito V Serra S Brusa D Mazzola F Arruga F Vaisitti T . Extracellular nicotinamide phosphoribosyltransferase (NAMPT) promotes M2 macrophage polarization in chronic lymphocytic leukemia. Blood. (2015) 125:111–23. doi: 10.1182/blood-2014-07-589069 25368373

[52] Li M Kirtane AR Kiyokawa J Nagashima H Lopes A Tirmizi ZA . Local targeting of NAD+ salvage pathway alters the immune tumor microenvironment and enhances checkpoint immunotherapy in glioblastoma. Cancer Res. (2020) 80:5024–34. doi: 10.1158/0008-5472.can-20-1094 32998997 PMC7669613

[53] Numata Y Fujii T Toda C Okumura T Manabe T Takeda N . Digoxin promotes anoikis of circulating cancer cells by targeting Na+/K+-ATPase α3-isoform. Cell Death Dis. (2025) 16:1–12. doi: 10.1038/s41419-025-07703-z 40350473 PMC12066707

[54] Kurzeder C Doan Nguyen-Sträuli B Krol I Ring A Castro-Giner F Nüesch M . Digoxin for reduction of circulating tumor cell cluster size in metastatic breast cancer: a proof-of-concept trial. Nat Med. (2025) 31:1120–4. doi: 10.1038/s41591-024-03486-6 39856336 PMC12003195

[55] Digoxin disperses clusters of Malignant breast cancer cells | Inside precision medicine. In: Inside Precision Medicine. Inside Precision Medicine. Available online at: https://www.insideprecisionmedicine.com/topics/oncology/digoxin-disperses-clusters-of-malignant-breast-cancer-cells/ (Accessed July 7, 2025).

[56] Guchelaar HJ . Effect of digoxin on survival outcome in metastatic renal cell carcinoma patients treated with tyrosine kinase inhibitors. In: Center for Global Clinical Research Data. (2024) Available online at: https://vivli.org/effect-of-digoxin-on-survival-outcome-in-metastatic-renal-cell-carcinoma-patients-treated-with-tyrosine-kinase-inhibitors/ (Accessed July 7, 2025).

[57] Motzer RJ Escudier B Oudard S Hutson TE Porta C Bracarda S . Phase 3 trial of everolimus for metastatic renal cell carcinoma: Final results and analysis of prognostic factors. Cancer. (2010) 116:4256–65. doi: 10.1002/cncr.25219 20549832

[58] Ryan CW Tangen CM Heath EI Stein MN Meng MV Alva AS . Adjuvant everolimus after surgery for renal cell carcinoma (EVEREST): a double-blind, placebo-controlled, randomised, phase 3 trial. Lancet. (2023) 402:1043–51. doi: 10.1016/s0140-6736(23)00913-3 37524096 PMC10622111

[59] Homicsko K Lukashev A Iggo RD . RAD001 (Everolimus) improves the efficacy of replicating adenoviruses that target colon cancer. Cancer Res. (2005) 65:6882–90. doi: 10.1158/0008-5472.can-05-0309 16061672

[60] Wu B Zhang B Li B Wu H Jiang M . Cold and hot tumors: from molecular mechanisms to targeted therapy. Signal Transduction Targeted Ther. (2024) 9:1–65. doi: 10.1038/s41392-024-01979-x 39420203 PMC11491057

[61] Betts MR Brenchley JM Price DA De Rosa SC Douek DC Roederer M . Sensitive and viable identification of antigen-specific CD8+ T cells by a flow cytometric assay for degranulation. J Immunol Methods. (2003) 281:65–78. doi: 10.1016/s0022-1759(03)00265-5 14580882

[62] Jubel JM Barbati ZR Burger C Wirtz DC Schildberg FA . The role of PD-1 in acute and chronic infection. Front Immunol. (2020) 11:524474. doi: 10.3389/fimmu.2020.00487 32265932 PMC7105608

[63] Stenger TD Miller JS . Therapeutic approaches to enhance natural killer cell cytotoxicity. Front Immunol. (2024) 15:1356666. doi: 10.3389/fimmu.2024.1356666 38545115 PMC10966407

[64] Hsu J Hodgins JJ Marathe M Nicolai CJ Bourgeois-Daigneault MC Trevino TN . Contribution of NK cells to immunotherapy mediated by PD-1/PD-L1 blockade. J Clin Invest. (2018) 128:4654–68. doi: 10.1172/jci99317 30198904 PMC6159991

[65] Liu Y Cheng Y Xu Y Wang Z Du X Li C . Increased expression of programmed cell death protein 1 on NK cells inhibits NK-cell-mediated anti-tumor function and indicates poor prognosis in digestive cancers. Oncogene. (2017) 36:6143–53. doi: 10.1038/onc.2017.209 28692048 PMC5671935

[66] Concha-Benavente F Kansy B Moskovitz J Moy J Chandran U Ferris RL . PD-L1 mediates dysfunction in activated PD-1 þ NK cells in head and neck cancer patients. Cancer Immunol Res. (2018) 6:1548–60. doi: 10.1158/2326-6066.cir-18-0062 30282672 PMC6512340

[67] Tong L Tay AHM Cui W Liu Y Su Y Lyu J . VHL restoration in clear cell renal cell carcinoma improves NK cell infiltration and function. Cancer Immunol Immunother. (2025) 74:1–14. doi: 10.1007/s00262-025-04132-x 40736709 PMC12311085

