# Supplementary material for: Novel therapeutic strategies for ccRCC through integration of systemic drug combinations and oncolytic adenovirus immunotherapy
Source: Front Immunol. 2026 Aug 14;17:1760889. doi: 10.3389/fimmu.2026.1760889 (PMC13522144; doi:10.3389/fimmu.2026.1760889)
Supplement: Supplementary file 1 [file Table1.docx]

Supplementary Material

# Supplementary Tables

**Supplementary Table 1**

| **Patient ID** | **WHO at diagnosis** | **Operation technique** | **Histology** | **Grade** | **TNM pathology** | **T stage** | **Leibovich score risk group** | **Metastatic disease** | **TMB (coding non-silent mutations/Mb)** |
| --- | --- | --- | --- | --- | --- | --- | --- | --- | --- |
| Patient 1 | 0 | Nephrectomy, open | ccRCC | 4 | pT3aNXM1 | pT3 | High-risk | Yes, at the time of diagnosis | 1.8 |
| Patient 2 | 1 | Nephrectomy, open | ccRCC | 3 | pT3cN0M0 | pT4 | High-risk | Yes, < 2 years post diagnosis | 2.8 |
| Patient 3 | 0 | Nephrectomy, open | ccRCC | 3 | pT3bN0M1 | pT4 | High-risk | Yes, at the time of diagnosis | 2.6 |

# Supplementary Table 1. Clinical and pathological characteristics of Patient 1, Patient 2 and Patient 3.

**Supplementary Table 2**

| **Compound name** | **Solvent** | **Max concentration (nM) in 25 µl** | **Oncology status** | **Oncology status source** |
| --- | --- | --- | --- | --- |
| (-)-Ketoconazole | DMSO | 10000 | FDA: Approved (non-oncology) -> CT.gov: Investigational (Ph 3) |  |
| 1'2-Bis(4-bromophenyl)ethane-1'2-dione | DMSO | 10000 | No oncology evidence |  |
| 2'4-Dinitrophenol | DMSO | 10000 | No oncology evidence |  |
| 3-Bromopyruvic acid | AQ | 10000 | No oncology evidence |  |
| 3-Hydroxybutyric acid | AQ | 10000 | Investigational (Ph 2) | CT.gov |
| 3'5-Dichloro-2-hydroxybenzoic acid | DMSO | 10000 | No oncology evidence |  |
| 4-Nitrobenzenesulfonamide | DMSO | 10000 | No oncology evidence |  |
| 4H-Thieno[3'2-b]pyrrole-5-carboxylic acid | DMSO | 10000 | Not found |  |
| 5-Fluorouracil | DMSO | 10000 | Approved (oncology) | FDA |
| A 1070722 | DMSO | 10000 | No oncology evidence |  |
| AACOCF3 | DMSO | 10000 | No oncology evidence |  |
| Abiraterone | DMSO | 10000 | Approved (oncology) | FDA/EMA |
| Acarbose | AQ | 10000 | FDA: Approved (non-oncology) -> CT.gov: Investigational (Ph 2) |  |
| Acetylcysteine | DMSO | 10000 | FDA: Approved (non-oncology) -> CT.gov: Investigational (Ph 3) |  |
| AGI-5198 | DMSO | 10000 | No oncology evidence |  |
| Alpha-Naphthoflavone | DMSO | 10000 | No oncology evidence |  |
| Amitriptyline | DMSO | 10000 | FDA: Approved (non-oncology) -> CT.gov: Investigational (Ph 3) |  |
| Anastrozole | DMSO | 10000 | Approved (oncology) | FDA |
| AR-A014418 | DMSO | 10000 | No oncology evidence |  |
| Arginase inhibitor 1 | DMSO | 10000 | Investigational (Ph 2) | CT.gov |
| Arsenic trioxide | AQ | 10000 | Approved (oncology) | FDA/EMA |
| Aurintricarboxylic acid | DMSO | 10000 | No oncology evidence |  |
| AVN-944 | DMSO | 10000 | Investigational (Ph 2) | CT.gov |
| AZD3965 | DMSO | 10000 | Investigational (Ph 1) | CT.gov |
| Baicalein | DMSO | 10000 | No oncology evidence |  |
| Benzbromarone | DMSO | 10000 | No oncology evidence |  |
| Berberine | DMSO | 10000 | Investigational (Ph 3) | CT.gov |
| BMS-303141 | DMSO | 10000 | No oncology evidence |  |
| BrBzGCp2 | DMSO | 10000 | No oncology evidence |  |
| Brequinar | DMSO | 10000 | Investigational (Ph 2) | CT.gov |
| Bromoenol lactone | DMSO | 10000 | No oncology evidence |  |
| BVT 2733 | DMSO | 10000 | No oncology evidence |  |
| CaCl2 | AQ | 10000 | No oncology evidence |  |
| Capecitabine | DMSO | 10000 | Approved (oncology) | FDA/EMA |
| Carmustine | DMSO | 10000 | Approved (oncology) | FDA/EMA |
| Carnosic acid | DMSO | 10000 | No oncology evidence |  |
| Castanospermine | DMSO | 10000 | No oncology evidence |  |
| CBR-5884 | DMSO | 10000 | No oncology evidence |  |
| Celecoxib | DMSO | 10000 | FDA: Approved (non-oncology) -> CT.gov: Investigational (Ph 3) |  |
| CHC | DMSO | 10000 | Investigational (Ph 3) | CT.gov |
| Chloroquine | DMSO | 10000 | FDA: Approved (non-oncology) -> CT.gov: Investigational (Ph 3) |  |
| Cladribine | DMSO | 10000 | Approved (oncology) | FDA/EMA |
| Coenzyme A | DMSO | 10000 | Investigational (Ph 3) | CT.gov |
| CP-640186 | DMSO | 10000 | No oncology evidence |  |
| CPI-0610 | DMSO | 10000 | Investigational (Ph 3) | CT.gov |
| Cyclosporin A | DMSO | 10000 | EMA: Approved (non-oncology) -> CT.gov: Investigational (Ph 3) |  |
| Cytarabine | DMSO | 10000 | Approved (oncology) | FDA |
| Daidzin | DMSO | 10000 | No oncology evidence |  |
| Dapagliflozin | DMSO | 10000 | FDA/EMA: Approved (non-oncology) -> CT.gov: Investigational (Ph 3) |  |
| Daporinad | DMSO | 10000 | Investigational (Ph 2) | CT.gov |
| Darapladib | DMSO | 10000 | No oncology evidence |  |
| Deferoxamine | DMSO | 10000 | FDA: Approved (non-oncology) -> CT.gov: Investigational (Ph 3) |  |
| Devimistat | DMSO | 10000 | Investigational (Ph 3) | CT.gov |
| Diclofenac | DMSO | 10000 | FDA: Approved (non-oncology) -> CT.gov: Investigational (Ph 3) |  |
| Dicoumarol | DMSO | 10000 | No oncology evidence |  |
| Digoxin | DMSO | 10000 | FDA: Approved (non-oncology) -> CT.gov: Investigational (Ph 3) |  |
| Dipyridamole | DMSO | 10000 | FDA: Approved (non-oncology) -> CT.gov: Investigational (Ph 2) |  |
| Disulfiram | DMSO | 10000 | FDA: Approved (non-oncology) -> CT.gov: Investigational (Ph 3) |  |
| DL-Alanine | DMSO | 10000 | Investigational (Ph 3) | CT.gov |
| Dorsomorphin | DMSO | 10000 | No oncology evidence |  |
| Doxycycline | DMSO | 10000 | FDA: Approved (non-oncology) -> CT.gov: Investigational (Ph 3) |  |
| Ebselen | DMSO | 10000 | Investigational (Ph 2) | CT.gov |
| Econazole | DMSO | 10000 | Approved (non-oncology) | FDA |
| Ellagic acid | DMSO | 10000 | Investigational (Ph 3) | CT.gov |
| Emodin | DMSO | 10000 | No oncology evidence |  |
| Enasidenib | DMSO | 10000 | Approved (oncology) | FDA |
| Epalrestat | DMSO | 10000 | Investigational (Ph 2) | CT.gov |
| Etomidate | DMSO | 10000 | FDA: Approved (non-oncology) -> CT.gov: Investigational (Ph 3) |  |
| Everolimus | DMSO | 10000 | Approved (oncology) | FDA/EMA |
| Fadrozole | DMSO | 10000 | Investigational (Ph 2) | CT.gov |
| Fatostatin | DMSO | 10000 | No oncology evidence |  |
| FCCP | DMSO | 10000 | Investigational (Ph 3) | CT.gov |
| Febuxostat | DMSO | 10000 | Approved (oncology) | EMA |
| FIPI | DMSO | 10000 | No oncology evidence |  |
| Flavoxate (hydrochloride) | AQ | 10000 | Approved (non-oncology) | FDA |
| Fludarabine | DMSO | 10000 | Approved (oncology) | FDA |
| Gemcitabine | DMSO | 10000 | Approved (oncology) | FDA |
| GKT136901 | DMSO | 10000 | No oncology evidence |  |
| Glycyrrhizic acid | DMSO | 10000 | Investigational (Ph 3) | CT.gov |
| Gossypol | DMSO | 10000 | Investigational (Ph 3) | CT.gov |
| GSK2194069 | DMSO | 10000 | Investigational (Ph 3) | CT.gov |
| GW 4064 | DMSO | 10000 | No oncology evidence |  |
| GW9508 | DMSO | 10000 | No oncology evidence |  |
| GW9662 | DMSO | 10000 | No oncology evidence |  |
| Hydroxychloroquine (sulfate) | AQ | 10000 | FDA: Approved (non-oncology) -> CT.gov: Investigational (Ph 2) |  |
| Idoxuridine | DMSO | 10000 | Investigational (Ph 2) | CT.gov |
| Indisulam | DMSO | 10000 | Investigational (Ph 2) | CT.gov |
| Indomethacin | DMSO | 10000 | FDA: Approved (non-oncology) -> CT.gov: Investigational (Ph 3) |  |
| Indoximod | AQ | 10000 | Investigational (Ph 2) | CT.gov |
| Isocarboxazid | DMSO | 10000 | Approved (non-oncology) | FDA |
| Ivosidenib | DMSO | 10000 | Approved (oncology) | FDA/EMA |
| JZL 184 | DMSO | 10000 | No oncology evidence |  |
| Ketoprofen | DMSO | 10000 | FDA: Approved (non-oncology) -> CT.gov: Investigational (Ph 3) |  |
| L-Ascorbic acid | DMSO | 10000 | Investigational (Ph 3) | CT.gov |
| L-Glutathione reduced | AQ | 10000 | Investigational (Ph 3) | CT.gov |
| L-Kynurenine | DMSO | 10000 | Investigational (Ph 3) | CT.gov |
| Lestaurtinib | DMSO | 10000 | Investigational (Ph 3) | CT.gov |
| Lonidamine | DMSO | 10000 | No oncology evidence |  |
| Lovastatin | DMSO | 10000 | FDA: Approved (non-oncology) -> CT.gov: Investigational (Ph 3) |  |
| LY294002 | DMSO | 10000 | Investigational (Ph 1) | CT.gov |
| Mardepodect | DMSO | 10000 | No oncology evidence |  |
| MB05032 | DMSO | 10000 | No oncology evidence |  |
| MEDICA16 | DMSO | 10000 | No oncology evidence |  |
| Mercaptopurine | DMSO | 10000 | Approved (oncology) | FDA/EMA |
| Metformin (hydrochloride) | DMSO | 10000 | FDA: Approved (non-oncology) -> CT.gov: Investigational (Ph 3) |  |
| Methotrexate | DMSO | 10000 | Approved (oncology) | FDA/EMA |
| Methylene blue | AQ | 10000 | FDA: Approved (non-oncology) -> CT.gov: Investigational (Ph 3) |  |
| Methylmalonic acid | DMSO | 10000 | Investigational (Ph 2) | CT.gov |
| Metyrosine | AQ | 10000 | FDA: Approved (non-oncology) -> CT.gov: Investigational (Ph 3) |  |
| MF-438 | DMSO | 10000 | No oncology evidence |  |
| Miglitol | DMSO | 10000 | Approved (non-oncology) | FDA |
| MK-8245 | DMSO | 10000 | No oncology evidence |  |
| MKT-077 | DMSO | 10000 | No oncology evidence |  |
| Mycophenolic acid | DMSO | 10000 | FDA: Approved (non-oncology) -> CT.gov: Investigational (Ph 3) |  |
| N-Arachidonyl maleimide | DMSO | 10000 | No oncology evidence |  |
| N-Benzylformamide | DMSO | 10000 | No oncology evidence |  |
| N'N-Dimethylsphingosine | DMSO | 10000 | No oncology evidence |  |
| NHI-2 | DMSO | 10000 | No oncology evidence |  |
| Niacin | DMSO | 10000 | FDA: Approved (non-oncology) -> CT.gov: Investigational (Ph 3) |  |
| Nordihydroguaiaretic acid | DMSO | 10000 | Investigational (Ph 2) | CT.gov |
| Oligomycin A | DMSO | 10000 | No oncology evidence |  |
| Orlistat | DMSO | 10000 | Approved (oncology) | FDA |
| Papaverine | DMSO | 10000 | Investigational (Ph 3) | CT.gov |
| Pemetrexed | DMSO | 10000 | Approved (oncology) | FDA/EMA |
| Pentostatin | DMSO | 10000 | Approved (oncology) | FDA |
| Pepstatin | DMSO | 10000 | No oncology evidence |  |
| Perphenazine | DMSO | 10000 | Approved (non-oncology) | FDA |
| PF-543 | DMSO | 10000 | No oncology evidence |  |
| Pictilisib | DMSO | 10000 | Investigational (Ph 2) | CT.gov |
| Piperlongumine | DMSO | 10000 | No oncology evidence |  |
| Pirinixic acid | DMSO | 10000 | No oncology evidence |  |
| PluriSIn 1 | DMSO | 10000 | No oncology evidence |  |
| Polymyxin B | DMSO | 10000 | FDA: Approved (non-oncology) -> CT.gov: Investigational (Ph 3) |  |
| Prednisolone | DMSO | 10000 | Approved (oncology) | FDA |
| Raloxifene | DMSO | 10000 | Approved (oncology) | FDA |
| Raltitrexed | DMSO | 10000 | Investigational (Ph 3) | CT.gov |
| Rasagiline | DMSO | 10000 | Approved (non-oncology) | FDA/EMA |
| RHC 80267 | DMSO | 10000 | No oncology evidence |  |
| Ribavirin | DMSO | 10000 | FDA: Approved (non-oncology) -> CT.gov: Investigational (Ph 3) |  |
| Ritonavir | DMSO | 10000 | FDA/EMA: Approved (non-oncology) -> CT.gov: Investigational (Ph 2) |  |
| Ro 48-8071 | DMSO | 10000 | No oncology evidence |  |
| Rosiglitazone | DMSO | 10000 | Investigational (Ph 3) | CT.gov |
| SAH | DMSO | 10000 | Approved (oncology) | FDA |
| SB 204990 | DMSO | 10000 | No oncology evidence |  |
| SC-26196 | DMSO | 10000 | No oncology evidence |  |
| SC-57461A | DMSO | 10000 | No oncology evidence |  |
| Silibinin | DMSO | 10000 | Investigational (Ph 3) | CT.gov |
| Simvastatin | DMSO | 10000 | FDA: Approved (non-oncology) -> CT.gov: Investigational (Ph 3) |  |
| Sitagliptin | DMSO | 10000 | FDA/EMA: Approved (non-oncology) -> CT.gov: Investigational (Ph 3) |  |
| SKI II | DMSO | 10000 | Investigational (Ph 3) | CT.gov |
| Spautin-1 | DMSO | 10000 | No oncology evidence |  |
| SR12813 | DMSO | 10000 | No oncology evidence |  |
| Sulfasalazine | DMSO | 10000 | FDA: Approved (non-oncology) -> CT.gov: Investigational (Ph 3) |  |
| Tadalafil | DMSO | 10000 | FDA/EMA: Approved (non-oncology) -> CT.gov: Investigational (Ph 3) |  |
| Tanshinone I | DMSO | 10000 | Investigational (Ph 3) | CT.gov |
| TAS-102 | DMSO | 10000 | Approved (oncology) | FDA |
| Telaglenastat | DMSO | 10000 | Investigational (Ph 2) | CT.gov |
| Temsirolimus | DMSO | 10000 | Approved (oncology) | FDA/EMA |
| TEPP-46 | DMSO | 10000 | No oncology evidence |  |
| Terbinafine | DMSO | 10000 | FDA: Approved (non-oncology) -> CT.gov: Investigational (Ph 2) |  |
| Teriflunomide | DMSO | 10000 | FDA/EMA: Approved (non-oncology) -> CT.gov: Investigational (Ph 2) |  |
| Tigecycline | DMSO | 10000 | FDA/EMA: Approved (non-oncology) -> CT.gov: Investigational (Ph 2) |  |
| TMS | DMSO | 10000 | Approved (oncology) | FDA/EMA |
| TOFA | DMSO | 10000 | FDA: Approved (non-oncology) -> CT.gov: Investigational (Ph 3) |  |
| Tolrestat | DMSO | 10000 | No oncology evidence |  |
| Topotecan | DMSO | 10000 | Approved (oncology) | FDA/EMA |
| Torcetrapib | DMSO | 10000 | No oncology evidence |  |
| Torin 1 | DMSO | 10000 | Investigational (Ph 3) | CT.gov |
| trans-C75 | DMSO | 10000 | No oncology evidence |  |
| Triclosan | DMSO | 10000 | Investigational (Ph 3) | CT.gov |
| Trifluoperazine | AQ | 10000 | Approved (non-oncology) | FDA |
| Trimetazidine | DMSO | 10000 | Investigational (Ph 3) | CT.gov |
| UK-5099 | DMSO | 10000 | No oncology evidence |  |
| UNC0224 | DMSO | 10000 | No oncology evidence |  |
| URB597 | DMSO | 10000 | No oncology evidence |  |
| Valinomycin | DMSO | 10000 | No oncology evidence |  |
| Valproic acid | DMSO | 10000 | FDA: Approved (non-oncology) -> CT.gov: Investigational (Ph 3) |  |
| Varespladib | DMSO | 10000 | No oncology evidence |  |
| Voglibose | DMSO | 10000 | No oncology evidence |  |
| WWL113 | DMSO | 10000 | No oncology evidence |  |
| WZB117 | DMSO | 10000 | No oncology evidence |  |
| Zoledronic Acid | DMSO | 10000 | Approved (oncology) | FDA/EMA |

**Supplementary Table 2.** List of all 182 compounds, including their solvent, maximum concentration used in the DSRT, oncology status, and the source of the oncology status classification. Compounds are listed in alphabetical order.

**Supplementary Table 3**

| **Gene name** | **Patient 1** | **Patient 2** | **Patient 3** | **Gene name** | **Patient 1** | **Patient 2** | **Patient 3** | **Gene name** | **Patient 1** | **Patient 2** | **Patient 3** |
| --- | --- | --- | --- | --- | --- | --- | --- | --- | --- | --- | --- |
| ABCA10 | 0.056 | NA | NA | HS3ST4 | NA | 0.507 | NA | RERGL | 0.147 | NA | NA |
| ABCB11 | NA | NA | 0.285 | HSD17B11 | NA | 0.402 | NA | RETSAT | NA | NA | 0.098 |
| ABCC5 | NA | NA | 0.085 | HSF1 | NA | 0.232 | NA | RNF111 | NA | NA | 0.248 |
| ACAN | NA | 0.278 | NA | HTR4 | NA | 0.479 | NA | RNF19B | 0.118 | NA | NA |
| ACKR3 | NA | NA | 0.274 | ICE1 | 0.052 | NA | NA | RPS6 | NA | 0.073 | NA |
| ACOXL | 0.097 | NA | NA | IER5 | NA | 0.169 | NA | RSAD2 | NA | NA | 0.147 |
| ACTN4 | NA | NA | 0.327 | IFNLR1 | NA | 0.188 | NA | RTEL1 | NA | NA | 0.308 |
| ADAM10 | NA | NA | 0.229 | IRS2 | 0.081 | NA | NA | RUNX2 | NA | 0.272 | NA |
| ADRA1D | NA | NA | 0.154 | ITGB8 | NA | NA | 0.126 | SDF4 | NA | NA | 0.362 |
| ALPK1 | NA | NA | 0.179 | KCNC2 | NA | 0.595 | NA | SERPINA6 | NA | NA | 0.051 |
| ANK2 | NA | 0.385 | NA | KCNJ11 | NA | NA | 0.191 | SETD2 | NA | 0.568 | 0.365 |
| ANKRD24 | NA | NA | 0.25 | KDM4A | NA | NA | 0.105 | SETMAR | NA | 0.688 | NA |
| ANXA9 | NA | NA | 0.2 | KDM5B | 0.126 | NA | NA | SF1 | 0.139 | NA | NA |
| AP5Z1 | NA | NA | 0.197 | KDM5C | NA | 0.757 | NA | SGK1 | NA | 0.296 | NA |
| APOA1 | NA | 0.383 | NA | KIF18A | 0.059 | NA | NA | SHF | NA | 0.266 | NA |
| AQP1 | 0.091 | NA | NA | KIF19 | 0.054 | NA | NA | SIN3A | NA | NA | 0.393 |
| ARAP3 | NA | NA | 0.197 | LAMA1 | NA | 0.509 | NA | SIPA1L2 | NA | NA | 0.367 |
| AREG | NA | 0.22 | NA | LAMA2 | NA | 0.216 | NA | SLC15A4 | NA | NA | 0.311 |
| ARHGAP27 | NA | NA | 0.162 | LCA5L | NA | NA | 0.25 | SLC1A6 | NA | 0.181 | NA |
| ARHGAP32 | NA | 0.333 | NA | LDB3 | NA | 0.115 | NA | SLC25A25 | NA | 0.324 | NA |
| B4GALNT3 | NA | 0.193 | NA | LGR6 | 0.117 | NA | NA | SLC26A4 | NA | 0.44 | NA |
| BRD8 | 0.094 | NA | NA | LIPE | NA | NA | 0.088 | SLC2A1 | NA | 0.113 | NA |
| CABYR | 0.067 | NA | NA | LRRC1 | 0.153 | NA | NA | SLC39A10 | NA | NA | 0.222 |
| CDC14C | NA | 0.378 | NA | LRRC25 | NA | NA | 0.296 | SLC9A8 | 0.108 | NA | NA |
| CDH19 | NA | 0.272 | NA | MADD | NA | NA | 0.271 | SLFN13 | 0.056 | NA | NA |
| CDHR5 | NA | NA | 0.079 | MAP3K1 | 0.098 | NA | NA | SMARCA4 | 0.118 | NA | NA |
| CEBPZ | 0.083 | NA | NA | MARCO | 0.077 | NA | NA | SNRNP27 | NA | 0.317 | NA |
| CENPO | 0.051 | NA | NA | MED14OS | NA | 0.768 | NA | SON | NA | NA | 0.159 |
| CEP68 | NA | 0.407 | NA | METTL25 | NA | 0.064 | NA | SOWAHC | NA | 0.088 | NA |
| CNKSR2 | NA | 0.741 | NA | MRPS28 | 0.215 | NA | NA | SOX9 | NA | NA | 0.313 |
| CNTNAP3 | NA | NA | 0.156 | MRTO4 | NA | NA | 0.155 | SPEF2 | NA | 0.255 | NA |
| COL11A1 | NA | NA | 0.203 | MS4A15 | NA | NA | 0.293 | SPTBN5 | NA | NA | 0.262 |
| COL17A1 | NA | NA | 0.156 | MTFR2 | NA | NA | 0.307 | STAB1 | NA | 0.518 | NA |
| COL21A1 | NA | 0.284 | NA | MYH6 | 0.124 | 0.189 | NA | STARD9 | NA | 0.246 | NA |
| COL23A1 | 0.115 | NA | NA | MYLIP | NA | NA | 0.348 | STEAP2 | NA | 0.184 | NA |
| COL6A2 | NA | NA | 0.163 | MYO15A | NA | NA | 0.239 | SYNE1 | 0.062 | NA | NA |
| CORO1B | NA | NA | 0.355 | MYO7A | NA | NA | 0.308 | TAS2R5 | NA | 0.437 | NA |
| CTCF | NA | 0.208 | NA | MYT1L | 0.059 | NA | NA | TELO2 | 0.06 | NA | NA |
| CYP4V2 | NA | NA | 0.387 | NCAPG2 | NA | 0.075 | NA | TENM1 | NA | NA | 0.174 |
| DCHS2 | NA | 0.479 | NA | NCCRP1 | NA | 0.108 | NA | THAP12 | NA | NA | 0.308 |
| DDX20 | 0.097 | NA | NA | NEUROG3 | NA | NA | 0.133 | TM2D3 | 0.138 | NA | NA |
| DDX56 | 0.077 | NA | NA | NKX2-3 | 0.093 | NA | NA | TMCC3 | NA | 0.536 | NA |
| DGKZ | NA | 0.568 | NA | NLRP7 | NA | NA | 0.098 | TMEM132B | 0.109 | NA | NA |
| DHX35 | NA | 0.142 | NA | NLRP9 | NA | NA | 0.272 | TMEM236 | NA | NA | 0.176 |
| DNAH9 | NA | NA | 0.284 | ODF4 | NA | 0.252 | NA | TMX2 | NA | NA | 0.19 |
| DSEL | NA | NA | 0.344 | OR13H1 | NA | NA | 0.121 | TRPC4 | NA | NA | 0.114 |
| ERCC6L | NA | NA | 0.542 | OR5M3 | 0.092 | NA | NA | TTN | NA | 0.397 | NA |
| ESR2 | NA | NA | 0.533 | PACC1 | NA | NA | 0.111 | UBAC1 | NA | 0.293 | NA |
| FAR2 | 0.07 | NA | NA | PARP8 | 0.101 | NA | 0.176 | USP10 | 0.067 | NA | NA |
| FBXO24 | NA | 0.061 | NA | PBRM1 | NA | 0.646 | 0.293 | USP13 | NA | NA | 0.156 |
| FCN1 | NA | 0.23 | NA | PCDH15 | NA | 0.592 | NA | USP17L3 | NA | 0.576 | NA |
| FCRL6 | NA | 0.417 | NA | PCDHA5 | NA | NA | 0.18 | USP49 | NA | 0.368 | NA |
| FNIP2 | NA | 0.388 | NA | PDLIM2 | NA | 0.207 | NA | USP6 | 0.07 | NA | NA |
| GADD45A | 0.06 | NA | NA | PKD2 | NA | NA | 0.352 | VHL | 0.134 | 0.5 | 0.36 |
| GALNT11 | NA | NA | 0.336 | PLXNB1 | NA | NA | 0.452 | VIRMA | NA | NA | 0.282 |
| GALNT5 | NA | 0.409 | NA | PLXNB2 | 0.097 | 0.051 | NA | XRCC6 | NA | 0.398 | NA |
| GJC2 | NA | 0.304 | NA | PPP1CB | 0.128 | NA | NA | ZC3H12A | 0.053 | NA | NA |
| GLI3 | NA | 0.418 | NA | PRM1 | NA | NA | 0.315 | ZFPM2 | NA | 0.184 | NA |
| GPR17 | NA | NA | 0.108 | PRRC2B | NA | 0.275 | NA | ZIC3 | NA | 0.758 | NA |
| GPX4 | NA | 0.456 | NA | PRRX1 | 0.09 | NA | 0.33 | ZNF292 | 0.07 | NA | NA |
| GREB1 | NA | NA | 0.288 | PRX | NA | 0.201 | NA | ZNF462 | NA | 0.373 | NA |
| GRID2IP | NA | 0.208 | NA | PSMD1 | NA | 0.375 | NA | ZNF587B | NA | 0.072 | NA |
| GRK3 | 0.108 | NA | 0.347 | RAD17 | NA | NA | 0.431 | ZNF664-RFLNA | NA | 0.256 | NA |
| GTF2H1 | 0.239 | NA | NA | RASGRF1 | NA | NA | 0.247 | ZNF730 | NA | NA | 0.277 |
| HGS | NA | 0.516 | NA | RASGRP2 | NA | NA | 0.291 | ZNF800 | NA | NA | 0.327 |
| HMHB1 | 0.108 | NA | NA | RBFA | NA | 0.281 | NA | ZNF878 | NA | 0.378 | NA |
| HNRNPM | NA | NA | 0.247 | RBM14-RBM4 | NA | 0.479 | NA | ZNF99 | 0.068 | NA | NA |
| HS2ST1 | NA | NA | 0.138 | RBM39 | NA | 0.355 | NA | ZSCAN12 | NA | NA | 0.295 |

**Supplementary Table 3.** List of identified somatic mutations in the tumor tissue reporting the maximum variant allele frequency for Patient 1, Patient 2 and Patient 3. Genes are listed in alphabetical order.

**Supplementary Table 4**

| **Compound name** | **Solvent** | **Max concentration (nM) in 25 µl** |
| --- | --- | --- |
| Aurintricarboxylic acid | DMSO | 10000 |
| AVN-944 | DMSO | 10000 |
| CBR-5884 | DMSO | 10000 |
| CP-640186 | DMSO | 10000 |
| CPI-0610 | DMSO | 10000 |
| Cytarabine | DMSO | 10000 |
| Daporinad | DMSO | 10000 |
| Darapladib | DMSO | 10000 |
| Digoxin | DMSO | 10000 |
| Ebselen | DMSO | 10000 |
| Everolimus | DMSO | 10000 |
| GW 4064 | DMSO | 10000 |
| Lonidamine | DMSO | 10000 |
| Lovastatin | DMSO | 10000 |
| Methylene blue | AQ | 10000 |
| Pictilisib | DMSO | 10000 |
| Pirinixic acid | DMSO | 10000 |
| PluriSIn 1 | DMSO | 10000 |
| Polymyxin B | DMSO | 10000 |
| Simvastatin | DMSO | 10000 |
| Temsirolimus | DMSO | 10000 |
| Topotecan | DMSO | 10000 |
| Torcetrapib | DMSO | 10000 |
| Torin 1 | DMSO | 10000 |
| Trimetazidine | DMSO | 10000 |
| Valinomycin | DMSO | 10000 |

**Supplementary Table 4.** List of 26 compounds, their solvent and the maximum concentration used in the DSRT. Compounds are listed in alphabetical order.

**Supplementary Table 5**

| **Drug(s)** | **Target(s)** | **Target geneset(s)** |
| --- | --- | --- |
| Daporinad | NAMPT | HALLMARK_INFLAMMATORY_RESPONSE,  HALLMARK_INTERFERON_GAMMA_RESPONSE |
| Temsirolimus, Torin1, Everolimus | MTOR | REACTOME_ADAPTIVE_IMMUNE_SYSTEM |
| AVN944 | IMPDH1/2 | REACTOME_INFECTIOUS_DISEASE,  REACTOME_SARS_COV_INFECTIONS,  REACTOME_POTENTIAL_THERAPEUTICS_FOR_SARS,  REACTOME_INNATE_IMMUNE_SYSTEM,  REACTOME_NEUTROPHIL_DEGRANULATION |
| Digoxin | ATP1A1/2/3, ATP1B1/2/3, SLC04C1 | REACTOME_INFECTIOUS_DISEASE,  REACTOME_SARS_COV_INFECTIONS,  REACTOME_POTENTIAL_THERAPEUTICS_FOR_SARS,  REACTOME_INNATE_IMMUNE_SYSTEM,  REACTOME_NEUTROPHIL_DEGRANULATION |

**Supplementary Table 5.** Mapping of six drugs to immune-related target gene sets using the HALLMARK and REACTOME gene set collections.

**Supplementary Table 6**

| **Compound name** | **Min concentration tested** | **Max concentration tested** | **EC50_MOCK** | **Log EC50_MOCK** |
| --- | --- | --- | --- | --- |
| Valinomycin | 1 | 10000 | 69,15 | 1,84 |
| Daporinad | 1 | 10000 | 292,5 | 2,466 |
| Torin 1 | 1 | 10000 | 95,55 | 1,98 |
| Everolimus | 1 | 10000 | 7,134 | 0,8533 |
| Topotecan | 1 | 10000 | 1016 | 3,007 |
| Pictilisib | 1 | 10000 | 2027 | 3,307 |
| Temsirolimus | 1 | 10000 | 73,06 | 1,864 |
|  |  |  |  |  |
| **Compound name** | **Min concentration tested** | **Max concentration tested** | **EC50_AdCab** | **Log EC50_AdCab** |
| Daporinad | 1 | 10000 | 69,41 | 1,841 |
| Topotecan | 1 | 10000 | 304,9 | 2,484 |
| CBR-5884 | 1 | 10000 | 5,375E+15 | 15,73 |
| Methylene Blue | 1 | 10000 | 1197 | 3,078 |
| Valinomycin | 1 | 10000 | 1,425E+30 | 30,15 |

**Supplementary Table 6.** LogEC50 values calculated by PRISM software for top performing drugs in MOCK and AdCab infected 786-O cells.

## Supplementary Figures

## Supplementary Figure 1

**Supplementary Figure 1. Drug responses at DSS>7 for the uninfected and infected 786-O renal cancer cell line.** Dose-response curves of drugs targeting metabolic pathways in 786-O cells. Curves show average response values across increasing drug concentrations (1-lowest, 5-highest) for the top performing drugs in uninfected 786-O cells; valinomycin (A), daporinad (B), torin 1 (C), everolimus (D), topotecan (E), pictilisib (F), temsirolimus (G) and in AdCab infected 786-O cells; daporinad (H), topotecan (I), CBR-5884 (J), methylene blue (K) and valinomycin (L). Curves represent replicate screens (n=2), with concentration 0 corresponding to the DMSO negative control. Vertical lines indicate LogEC50 values calculated by Prism software when available (**Supplementary Table 6**).

**Supplementary Figure 2**

**Supplementary Figure 2.** **Synergy profiles of AdCab in combination with selected compounds across PDC models based on HSA synergy analysis.** Synergy analysis using the Highest Single Agent (HSA) model across daporinad, digoxin, valinomycin, CPI-0610, everolimus, and torin 1 revealed compound- and patient-specific interaction patterns. Daporinad, valinomycin, and everolimus exhibited positive synergy scores in selected PDC models, whereas digoxin, CPI-0610, and torin 1 showed predominantly negative HSA scores, indicating antagonistic interactions.

**Supplementary Figure 3**

**Supplementary Figure 3.** **Drug treatment elicits patient-specific responses under uninfected and infected conditions.** Separation of drug responses by patient and condition highlights patient-specific differences between uninfected and infected cells for compounds with a mean DSS ≥ 5 per patient per condition (A). Comparison of biological versus technical standard deviation (SD) of DSS values for all 26 compounds under MOCK (circles) and AdCab (triangles) conditions highlights compounds with a biological-to-technical SD ratio (signal-to-noise ratio) ≥1.5, indicating substantial inter-patient variability (B). Volcano plot summarizing the effect size (biological-to-technical SD ratio) and statistical significance (Kruskal-Wallis test) of patient-specific drug responses, with dashed lines indicating the significance threshold and the SD ratio cutoff (≥1.5) used to define significant heterogeneity (C). Three drugs, daporinad, digoxin and everolimus, selected based on their immune-related mechanism of action, demonstrated varying DSS-based efficacy across PDCs of patients 1-3 in both uninfected and AdCab infected conditions (D). Single-concentration dose-response curves for each selected compound and patient under MOCK and AdCab conditions (E-M) are shown, where a decreasing curve slope indicates reduced cell viability, and a flat or increasing slope indicates little or no effect on target cells.

**Supplementary Figure 4**

**Supplementary Figure 4. Representative flow cytometry gating strategy for the identification of activated CD8^+^ T-cells in PDC cultures.** Gating was performed separately for MOCK, AdCab, and AdCab plus drug-treated samples and used to quantify the geometric mean (GM) of CD8⁺ T cells (A) and PD1^+^ CD8⁺ T-cells (B). Antibodies used for gating were: FITC anti-human CD56 (NK cells), PE/Cyanine7 anti-human CD3 (T-cells), PE/Cyanine5 anti-human CD8 (T-cells), and Pacific Blue anti-human CD279 (PD-1 marker).

**Supplementary Figure 5**

**Supplementary Figure 5. Representative flow cytometry gating strategy for the detection of NK cell presence in PDC cultures.** The applied gating strategy is shown separately for MOCK, AdCab and AdCab plus drug treatment for the detection of PD1^+^ (A) and CD107a^+^ (B) NK cells in all PDC models. Antibodies used for gating were: FITC anti-human CD56 (NK cells), Alexa Fluor 700 anti-human CD3 (T-cells), Pacific Blue anti-human CD279 (PD-1 marker) and PerCP anti-human CD107a.

**Supplementary Figure 6**

**Supplementary Figure 6. Human immune cell interactions with PDCs in co-culture models.** Spatial dispersion of PDC spheroids of Patient 1 (A), Patient 2 (B) and Patient 3 (C) was quantified using the mean NND/sphere radius, with lower values corresponding to denser spheroid packing in uninfected spheroids and higher values corresponding to greater spheroid scattering following viral infection. Cell localization measured in the sphere center (D, E and F) and sphere periphery (G, H and I) showed an overall increase and respective decrease with AdCab and daporinad treatment. A trend towards increased PBMC and cancer cell interactions was observed following combination treatment with AdCab and daporinad in PDCs of Patient 1 (J) and Patient 2 (K) compared to PDCs of Patient 3 (L). Significance levels were set at *p < 0.05, **p < 0.01, ***p < 0.001 and ****p < 0.0001 (one-way ANOVA).
